# Genetic recombination-mediated evolutionary interactions between phages of potential industrial importance and prophages of their hosts within or across the domains of *Escherichia, Listeria*, *Salmonella, Campylobacter*, and *Staphylococcus*

**DOI:** 10.1186/s12866-024-03312-6

**Published:** 2024-05-04

**Authors:** Saba Kobakhidze, Stylianos Koulouris, Nata Kakabadze, Mamuka Kotetishvili

**Affiliations:** 1 Hygiene and Medical Ecology, G. Natadze Scientific-Research Institute of Sanitary, 78 D. Uznadze St. 0102, Tbilisi, Georgia; 2 Faculty of Medicine, Iv. Javakhishvili Tbilisi State University, 1 Ilia Chavchavadze Ave. 0179, Tbilisi, Georgia; 3 Directorate General for Health and Food Safety (DG-SANTE), European Commission, 1049 Bruxelles/Brussel, Belgium; 4 Scientific Research Institute, School of Science and Technology, the University of Georgia, 77a M. Kostava St., 0171 Tbilisi, Georgia

**Keywords:** Bacteriophage, Phage, Prophage, Genetic recombination, Lateral genetic transfer, Intergeneric recombination

## Abstract

**Background:**

The in-depth understanding of the role of lateral genetic transfer (LGT) in phage-prophage interactions is essential to rationalizing phage applications for human and animal therapy, as well as for food and environmental safety. This *in silico* study aimed to detect LGT between phages of potential industrial importance and their hosts.

**Methods:**

A large array of genetic recombination detection algorithms, implemented in SplitsTree and RDP4, was applied to detect LGT between various *Escherichia, Listeria, Salmonella, Campylobacter, Staphylococcus, Pseudomonas*, and *Vibrio* phages and their hosts. PHASTER and RAST were employed respectively to identify prophages across the host genome and to annotate LGT-affected genes with unknown functions. PhageAI was used to gain deeper insights into the life cycle history of recombined phages.

**Results:**

The split decomposition inferences (bootstrap values: 91.3–100; fit: 91.433-100), coupled with the Phi (0.0-2.836E-12) and RDP4 (*P* being well below 0.05) statistics, provided strong evidence for LGT between certain *Escherichia, Listeria, Salmonella*, and *Campylobacter* virulent phages and prophages of their hosts. The LGT events entailed mainly the phage genes encoding for hypothetical proteins, while some of these genetic loci appeared to have been affected even by intergeneric recombination in specific *E. coli* and *S. enterica* virulent phages when interacting with their host prophages. Moreover, it is shown that certain *L. monocytogenes* virulent phages could serve at least as the donors of the gene loci, involved in encoding for the basal promoter specificity factor, for *L. monocytogenes*. In contrast, the large genetic clusters were determined to have been simultaneously exchanged by many *S. aureus* prophages and some *Staphylococcus* temperate phages proposed earlier as potential therapeutic candidates (in their native or modified state). The above genetic clusters were found to encompass multiple genes encoding for various proteins, such as e.g., phage tail proteins, the capsid and scaffold proteins, holins, and transcriptional terminator proteins.

**Conclusions:**

It is suggested that phage-prophage interactions, mediated by LGT (including intergeneric recombination), can have a far-reaching impact on the co-evolutionary trajectories of industrial phages and their hosts especially when excessively present across microbially rich environments.

**Supplementary Information:**

The online version contains supplementary material available at 10.1186/s12866-024-03312-6.

## Background

Bacteriophages, also called phages, represent the most prevalent biological entities of the biosphere, having a marked impact on the functional diversity of various ecosystems [1, 2]. Phages can be classified according to their life cycle categories being virulent (lytic) versus lysogenic (temperate). Approximately 40–50% of bacterial genomes are suggested to carry prophages [3] sometimes referred to as “time bombs” [4]. Intact prophages represent temperate phages that reside either as chromosomal elements or autonomous plasmids across bacterial genomes. Both the intact and defective prophages have been important sources for lateral genetic transfer (LGT) [5], contributing to the emergence of pathogenicity, virulence, ecological fitness, and antimicrobial resistance in natural populations of human and/or animal pathogenic bacteria [6, 7].

Unlike temperate phages, virulent phages have been frequently used for human and animal therapy, as well as in food safety and environmental safety practices [8; 9]. The pros and cons of different phage formulations were discussed earlier, suggesting that the disadvantages of their use, for the above purposes, are minor [8]. However, it is noteworthy that the recent advances made in phage research have revealed some alarming trends exhibited by the implications of these viral organisms in different mammal diseases including human diseases [9–15]. For example, a shift in the gut phage community composition was stated to contribute to the shift from health to disease conditions in humans [15–17]. A quantity increase of lytic phages in the intestines of humans was found recently to correlate with certain dysbiosis-linked diseases [1]. It was shown that individuals with leukemic diseases [18], or with inflammatory bowel disease (IBD) [14], shed a higher number of EC-phages (phages present as extracellular particles) in their feces. Phage-induced LGT has been considered to be part of the molecular-genetic mechanisms contributing to the shift from a healthy to a diseased state in humans [16]. In this light, considering the recently proposed concept on phages as being human pathogens [10], there has been a great need for a more in-depth understanding of their evolutionary trends being mediated specifically by LGT.

It is suggested that gene shuffling between phages and prophages takes place regularly, and when being prevalent, prophages provide ample opportunities for multiple genetic exchanges to occur [19], affecting either or both these phages and their host. Importantly, LGT was found to contribute not only to the genetic divergence of these viral agents, but also to the natural exchange of lytic modules between virulent phages and prophages [20], drastically expanding their gene repertoires, and sometimes even transferring functional innovations across taxa [21]. Besides, it is thought that LGT between different phages and prophages or their DNA derivatives can occur more frequently than previously anticipated [22], and that virulent phages facilitate the transfer of genes reaching even genetically very distant temperate phages [21]. Hence, it is highly imperative to determine the evolutionary trajectories of virulent phages and their hosts mediated collectively by LGT-induced phage-prophage interactions to better rationalize phage formulations and their use, especially for human and animal therapies, as well as for food and environmental safety.

Our *in silico* study, employing a large panel of well-established genetic recombination detection algorithms, demonstrates LGT events, which could occur between potential therapeutic versus biocontrol candidate phages and prophages of their hosts from the genera of *Escherichia, Salmonella*, *Listeria, Campylobacter*, *Pseudomonas, Staphylococcus* and *Vibrio*. Here, we provide strong statistical evidence for LGT events, which could occur between certain *E. coli, S. enterica, L. monocytogenes*, and *C. jejuni* virulent phages and intact or defective prophages of their hosts. Events of intergeneric recombination were suggested to also occur via the above phage-prophage interactions in some of these *E. coli, S. enterica, L. monocytogenes* phages and their hosts. These LGT events were determined to entail predominantly the genes of unknown functions. Specifically, extensive Horizontal Gene Transfer (HGT) events were strongly suggested to have occur between certain *S. aureus* prophages and specific phages of this species most likely with temperate lifestyle, while being considered earlier to be an alternative (in their native or modified form) to antimicrobials for treating *Staphylococcus* infections [23–26]. Very importantly, when interacting with their host strains, these *S. aureus* phages were demonstrated to have abilities to obtain and/or to donate significantly large genomic regions in such LGT events, involving the genes with different functions, which encode for phage tail proteins, the capsid and scaffold proteins, holins, and some other important phage proteins. The trajectories and chromosomal coverages of all the above LGT events could be determined as well.

## Methods

### Phage and bacterial strains

The complete genomes, for a total of 101 phages infecting different host species, were examined in the genetic recombination analyses. A host spectrum of these organisms exhibited collectively *E. coli, L. monocytogenes, S. enterica, C. jejuni, C. coli, S. aureus*, as well as some *Pseudomonas* and *Vibrio* species. In this phage collection, all the phages were previously determined as virulent except the *S. aureus* phages SA75 (with an unknown life cycle) and JD419 (with a temperate life cycle). The nucleotide sequences of all the phages genomes were available in the nucleotide database of the National Center for Biotechnology Information (NCBI, https://www.ncbi.nlm.nih.gov/). The GenBank accession numbers, for the above phages, are provided in Table S1. The DNA sequences of bacterial genomic regions, exhibiting their homology to the phage chromosomal loci in the above database, were also included in the recombination analyses.

### Selection of phage and bacterial genomic homologs

The Basic Local Alignment Search Tool (BLAST) [27] with the megaBLAST algorithm was employed to determine and select the bacterial genomic regions illuminating their homology towards the genomic regions of 101 virulent phages in the NCBI database nucleotide collections (nr/nt). When using megaBLAST, the following default general and scoring parameters were applied: Expected threshold − 10/; Word size − 28; Max matches in a query range − 0; Match/mismatch scores − 1,-2; Gap costs – Linear; and Extension − 2. In the BLAST analyses, all regions of low compositional complexity were filtered. While using the very strict general and scoring parameters, especially with the word size being 28 in the above BLAST analysis, only the DNA sequences of bacterial genetic loci, exhibiting ≥ 90% DNA similarity with the phage genomic regions, were selected for the subsequent genetic recombination tests.

### Genetic recombination analyses

Initially, analyzing the BLAST-identified phage and bacterial genomic homologs, we applied the split decomposition method [28], implemented in the SplitsTree program (version 4.14.4) [29], to detect and reconstruct HGT events between these organisms. For these *in silico* analyses, the DNA sequences of the homologous genetic loci were aligned, using ClustalX (version 2.1) [30], and then, their ClustalX-generated alignments were analyzed by the above method. When detected, HGT events were displayed as a parallelogram(s) by SplitsTree. We performed bootstrapping (using 10 000 replicates) to assess the statistical robustness of the SplitsTree-generated inferences in these analyses. The bootstrap values ≥ 90 for the nodes of the parallelograms, when supported by the fit values being ≥ 95 for the entire splits networks, were considered to be statistically highly significant. The selected DNA sequence subsets, reflecting the HGT signals in the split decomposition analyses, were then subjected to the Phi (Pairwise Homoplasy Index) test [31]. Specifically, the Phi test was employed to detect false positive signals that could occur due to a possible presence of convergent mutations mimicking LGT events across the targeted genetic loci.

The DNA sequence alignments of the above homologous genetic loci that were exhibiting HGT signals in the SplitsTree analyses, were further reexamined by the RDP4 software package (Beta 4.96) [32]. In the RDP4 analyses, we applied the following recombination detection algorithms: RDP [33], GENECONV [34], BootScan [35], MaxChi [36], Chimaera [37], SiScan [38], and 3Seq [39]. These algorithms were used to determine the genetic recombination beginning and end breakpoints across the HGT-affected homologous loci, as well as the trajectories of the HGT events identified between phage and bacteria. While using RDP4, for the number of permutations, we used the default parameter – 0, the linear sequence setting, and the enabled disentangle recombination signals. The RDP4 analyses were conducted with a very stringent approach: When detected, for the significant breakpoint clusters (99%), only the predetermined Bonferroni-corrected *P-*values in a range of ≤ 0.05 were considered to be statistically significant.

### Prophage detection and phage life cycle-predictive *in silico* analyses

We used PHASTER (PHAge Search Tool-Enhanced Release, http://phaster.ca/) to determine whether the LGT-affected genomic regions belonged to intact or defective prophages in the targeted bacterial strains. In these analyses, PHASTER was applied as described previously [40, 41], using the following score ranges: > 90, 70–90, and < 70 for determining and classifying respectively intact, questionable, and defective prophages across the bacterial genomes. In addition, the RAST tool kit (RASTtk) [42] was employed to reannotate the recombined genes that encoded for hypothetical proteins (as recorded in the NCBI database), being our first step towards gaining initial insights into their functions. Besides, while the information on the life cycle of the virulent phages, involved in our study, was available in the respective literature and/or the NCBI GenBank records, we subsequently analyzed all the LGT-affected phage genomes using PhageAI (https://phage.ai/). PhageAI is the advanced machine learning and natural language processing software for the phage life cycle prediction, which, in our *in silico* analysis, was applied to gain some additional insights into the lifestyle evolution of these phages.

## Results

### Genetic recombination analyses of *Escherichia* and *Listeria* virulent phages

**Fig. 1:**
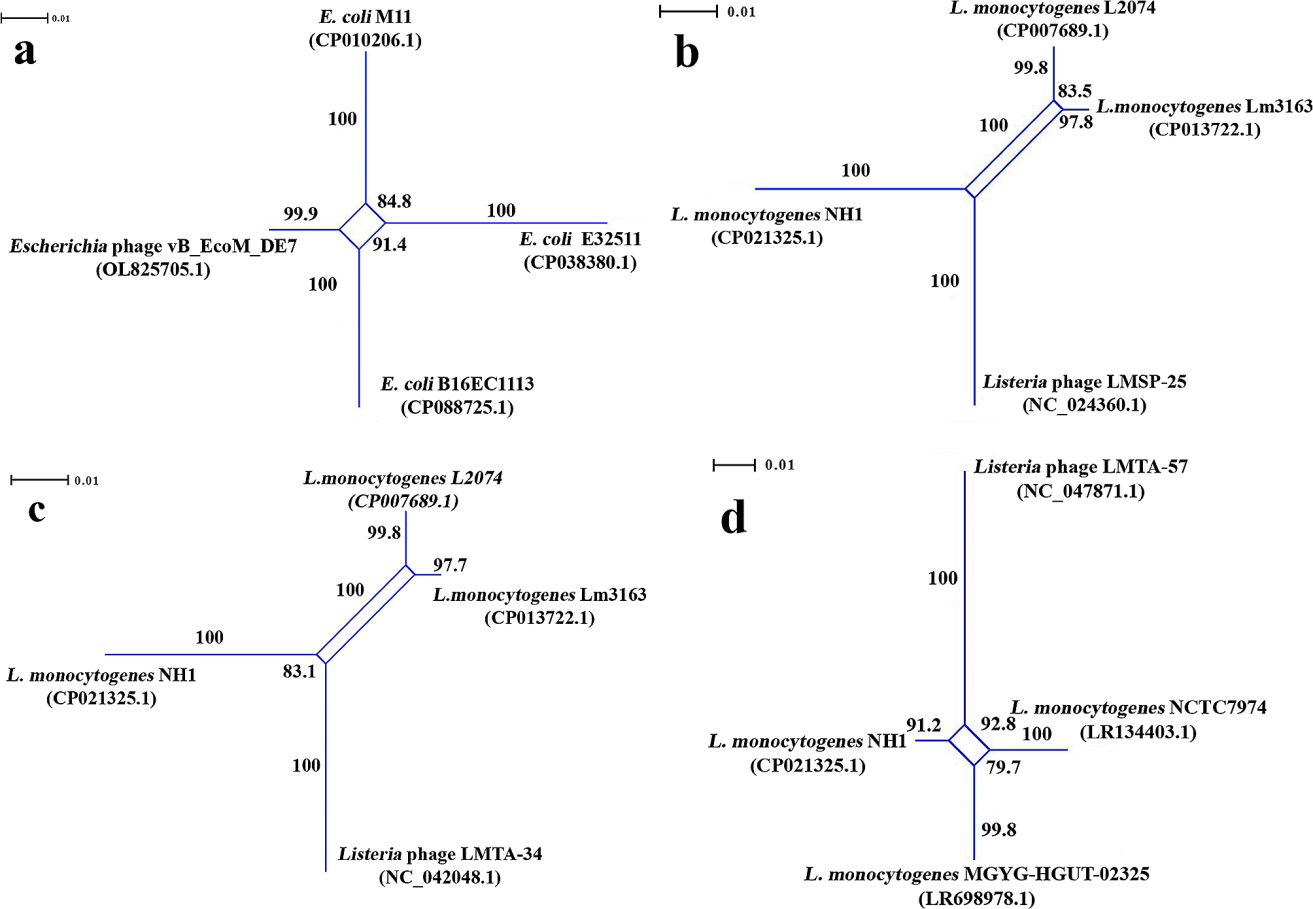
The SplitTree-generated parallelograms exhibiting conjointly the HGT events between the virulent *Escherichia* phage and some *E. coli* strains, and between three virulent *Listeria* phages and several *L. monocytogenes* strains. The HGT events identified between: (**a**) *Escherichia* phage vB_EcoM_DE7 (OL825705.1) and *E. coli* strains, when analyzing the phage 544-bp chromosomal region involved in encoding for the hypothetical protein (GenBank protein ID: UKH49269.1) (Fit: 100); (**b**) *Listeria* phage LMSP-25 (NC_024360.1) and *L. monocytogenes* strains, when analyzing the phage 684-bp chromosomal region involved in encoding for the hypothetical proteins (GenBank protein IDs: YP_009043028.1, YP_009043029.1, and YP_009043030.1), and DNA methyltransferase (GenBank protein ID: YP_009043031.1) (Fit: 100); (**c**) *Listeria* phage LMTA-34 (NC_042048.1) and *L. monocytogenes* strains, when analyzing the phage 684-bp chromosomal region involved in encoding for the hypothetical proteins (GenBank protein IDs: YP_009616146.1, YP_009616147.1., and YP_009616148.1) and DNA methyltransferase (GenBank protein ID: YP_009616149.1) (Fit: 100); (**d**) *Listeria* phage LMTA-57 (NC_047871.1) and *L. monocytogenes* strains, when analyzing the phage 684-bp chromosomal region involved in encoding for the hypothetical proteins (GenBank protein IDs: YP_009793497.1, YP_009793498.1, and YP_009793499.1) and DNA methyltransferase (GenBank protein ID: YP_009793496.1) (Fit: 100). In the splits graphs, the phage names appear according to their designations as presented in the NCBI GenBank database

Among twenty-nine *Escherichia* phages, we could identify only a single phage, vB_EcoM_DE7 (GenBank acc. # OL825705.1) that appeared to have exchanged genetic loci with two *E. coli* strains (the GenBank acc. #: CP088725.1 and CP010206). Specifically, using the split decomposition method, the SplitsTree analysis of the BLAST-determined 544-bp homologous genetic loci (the phage genome coordinates: 30376.30919) could generate a single parallelogram exhibiting the putative LGT events between the above organisms (Fig. 1a). As shown, the nodes of the parallelogram, shared by the above *Escherichia* phage and these *E. coli* strains, were supported by both the bootstrap estimates and the highest fit being 100. The split decomposition method-derived recombination signals (Fig. 1a) were confirmed by the Phi test-produced *p-*value 0.002507 (Table 1). In addition, we could determine the recombination beginning and end breakpoints across these homologous genetic loci, as well as the trajectories of the above LGT events, supported highly by the strong *p*-values when using MaxChi, SiScan, and 3Seq in the RDP4 analysis (Table 2). As shown, the recombined chromosomal region was associated with a gene encoding for a hypothetical protein (GenBank protein ID: UKH49269.1) in the *Escherichia* phage vB_EcoM_DE7. As determined by RDP4, in a single putative LGT event, this phage appeared to be a recombinant organism in contrast to the *E. coli* strains B16EC1113 (CP088725.1) and M11 (CP010206) serving respectively as its major and minor donors.

**Table 1:** The Phi test-generated probability (*p*) values obtained from the DNA sequences analyses of the recombined homologous genomic regions exchanged between the phage and bacterial strains as determined by the split decomposition method

SplitsTree-generated Splits graph	Phi test-generated *p*-value
Figure 1a	0.002507
Figure 1b	0.002079
Figure 1c	0.002079
Figure 1d	0.06912
Figure 2a	0.003484
Figure 2b	0.0191
Figure 2c	0.04491
Figure 2d	2.836E-12
Figure 3a	0.0
Figure 3b	0.0
Figure 3c	0.0

**Table 2:** The results of the RDP4 analyses exhibiting the recombination beginning and end breakpoints across the LGT-affected genetic loci, and the trajectories of the LGT events of these loci, involving the *Escherichia* phage and the *E. coli* strains versus the *Listeria* phages and *L. monocytogenes* strains

Recombinant strain (GenBank acc. #)	Major donor (GenBank acc. #)	Minor donor (GenBank acc. #)	CDS, for a protein, within a phage genomic region examined (Coordinates in a phage genome [size in bps])	Recombination beginning and end breakpoints (99% Cl) *	*P*-value generated by the RDP4 algorithm	
*Escherichia* phage vB_EcoM_DE7 (OL825705.1).	*E. coli* strain B16EC1113 (CP088725.1)	*E. coli* strain M11 (CP010206)	Hypothetical protein (ID: UKH49269.1) CDS: <1.>544. (30,376–30,919 [544])	308 (334 − 158) − 452 (334 − 158)	RDP: GENECONV: BootScan: MaxChi: Chimaera: SiScan: 3Seq:	- - - 1.139 × 10^− 03^ > 1.0 1.536 × 10^− 02^ 1.707 × 10^− 02^
*L. monocytogenes* strain NH1 (CP021325.1)	*L. monocytogenes* strain L2074 (CP007689.1)	*Listeria* phage LMSP-25 (NC_024360.1)	Hypothetical protein (ID: YP_009043029.1) CDS: 10.219. Hypothetical protein (ID: YP_009043030.1) CDS: 216.620. (21664.22347 [684])	176 (131–271) – 209 (131–271)	RDP: GENECONV: BootScan: MaxChi: Chimaera: SiScan: 3Seq:	9.876 × 10^− 03^ 1.959 × 10 − ^02^ 1.864 × 10^− 02^ 1.477 × 10^− 02^ 7.291 × 10^− 03^ 2.211 × 10^− 02^ -
*L. monocytogenes* strain NH1 (CP021325.1)	*L. monocytogenes* strain L2074 (CP007689.1)	*Listeria* phage LMTA-34 (NC_042048.1)	Hypothetical protein (ID: YP_009616147.1) CDS: 10.219. Hypothetical protein (ID: YP_009616148.1) CDS: 216.620. (21664.22347 [684])	176 (131–271) – 209 (131–271)	RDP: GENECONV: BootScan: MaxChi: Chimaera: SiScan: 3Seq:	9.876 × 10^− 03^ 1.959 × 10 − ^02^ 1.864 × 10^− 02^ 1.477 × 10^− 02^ 7.291 × 10^− 03^ 2.211 × 10^− 02^ -
*Listeria* phage LMTA-57 (NC_047871.1)	Unknown	*L. monocytogenes* strain NCTC7974 (LR134403.1)	Hypothetical protein (ID: YP_009793497.1) CDS: 65.469. (135369.136052 [684])	333 (229–370) – 412 (389–441)	RDP: GENECONV: BootScan: MaxChi: Chimaera: SiScan: 3Seq:	- 2.700 × 10^− 03^ - 4.551 × 10^− 03^ 3.944 × 10^− 03^ - 4.979 × 10^− 04^
*Listeria* phage LMTA-57 (NC_047871.1)	Unknown	*L. monocytogenes* strain NH1 (CP021325.1)	Hypothetical protein (ID: YP_009793497.1) CDS: 65.469. (135369.136052 [684])	176 (131–271) – 209 (131–271)	RDP: GENECONV: BootScan: MaxChi: Chimaera: SiScan: 3Seq:	4.938 × 10^− 02^ - - - 3.645 × 10^− 02^ 6.875 × 10^− 05^ -

CDS - Coding Sequence

Phages names appear according to their designations presented in the NCBI GenBank database

Recombination beginning and end breakpoints (99% Cl) * - The recombination beginning and end breakpoints in the DNA alignment

We applied PHASTER to determine whether the 544-bp genetic locus, putatively acquired by the above recombinant *Escherichia* phage, was associated with a possible prophage in these *E. coli* strains. PHASTER failed to identify any prophage region across the LGT-affected genetic loci in the genomes of the above *E. coli* strains. However, the hypothetical protein-encoding gene (UKH49269.1) was alternatively annotated by RAST as a phage gene encoding specifically for the Phage tail tape measure protein (TMP) (Table S2). In our analysis, the PhageAI-inferred life cycle (Table S3), for the *Escherichia* phage vB_EcoM_DE7, matched with (99.17% prediction accuracy) its actual life cycle being virulent as described previously [43].

The recombination analyses could identify three LGT-affected *Listeria* phages, LMSP-25 (NC_024360.1), LMTA-34 (NC_042048.1), and LMTA-57 (NC_047871.1), among eight virulent *Listeria* phages examined (Table S1). The putative LGT events, shared by these phages and some *L. monocytogenes*, were found to have involved certain genes encoding collectively for hypothetical proteins (YP_009043029.1, YP_009043030.1, YP_009616147.1, YP_009616148.1, YP_009793497.1, and YP_009793497.1) in the above viral agents. Specifically, in the initial genetic recombination analyses, the DNA sequences of the 684-bp homologous genomic regions of these *Listeria* phages and *L. monocytogenes* strains were analyzed using SplitsTree. The results obtained from the split decomposition analyses are presented in Fig. 1b-d, illuminating the putative LGT events between the *Listeria* virulent phages and the *L. monocytogenes* strains examined. As shown, a great majority of the nodes of the SplitsTree-constructed parallelograms, shared by the above organisms, were strongly supported by the bootstrap estimates and the highest fit value. When measuring homoplasy across the above-targeted homologous genetic loci, the Phi test resulted in the robust *p* estimate 0.002079 supporting strongly the LGT events presented in Fig. 1c-b. As shown, these putative LGT events entailed specifically the genetic interactions of the *Listeria* phages LMSP-25 (NC_024360.1) and LMTA-34 (NC_042048.1) with some *L. monocytogenes* strains. However, the Phi test-derived *p*-value 0.06912 (Table 1), received in the analysis of the targeted homologous genomic regions of the *Listeria* phage LMTA-57 (NC_047871.1) and three *L. monocytogenes*, was not supportive of the LGT signals reflected in the single parallelogram being shared by these organisms (Fig. 1d).

Using the RDP4-embeeded algorithms, we reexamined the DNA sequences of the 684-bp chromosomal regions of all the above *Listeria* phages and those of their respective homologs carried by the genomes of the *L. monocytogenes* strains in order to further assess the above LGT events. The recombination beginning and end breakpoints coupled with the extremely robust *p* estimates, obtained from the RDP4 analysis (Table 2), supported very strongly all the SplitsTree-detected LGT events shown in Fig. 1b-d. Both *Listeria* virulent phages, LMSP-25 (NC_024360.1) and LMTA-34 (NC_042048.1) were determined to be the minor donors of the internal loci of certain genes (encoding for the hypothetical proteins) for the *L. monocytogenes* strain NH1 (CP021325.1); as also shown in Table 2, RDP4 could determine additionally two independent putative LGT events. In these genetic recombination events, the *Listeria* phage LMTA-57 (NC_047871.1) served as a recombinant organism, while the *L. monocytogenes* strains NCTC7974 (LR134403.1) and NH1 (CP021325.1) appeared to be the minor donors of the internal loci of the same gene encoding for the hypothetical protein (YP_009793497.1). In our analysis, the LGT-affected genes, encoding for the hypothetical proteins under the GenBank IDs YP_009043029.1 and YP_009616147.1, were annotated by RAST as the protein gp55-encoding genes. In addition, the LGT-affected genes, involved in the synthesis of the hypothetical proteins under the GenBank IDs YP_009043030.1, YP_009616148.1, and YP_009793497.1, were designated by RAST generally as the genetic loci encoding for the phage protein (Table S2).

The PhageAI-generated life cycle inferences, exhibiting the prediction accuracy ranges of 93.69-93.81% (Table S3), for all the above recombining *Listeria* phages, strictly matched with the GenBank reports on their actual life cycle being virulent. In the genomes of the *L. monocytogenes* strains NH1 (CP021325.1) and NCTC7974 (LR134403.1), the LGT-affected bacterial homologs of the above phage genetic loci were associated with the intact prophages (the genome coordinates: 669954.706293, and 189,306…232,883 respectively) as determined by PHASTER (Table S4). Moreover, as shown, the above intact prophage of the *L. monocytogenes* strain NCTC7974 was found to represent an extrachromosomal circular plasmid in this organism (LR134403.1).

### Genetic recombination analyses of *Salmonella* and *Campylobacter* virulent phages

**Fig. 2:**
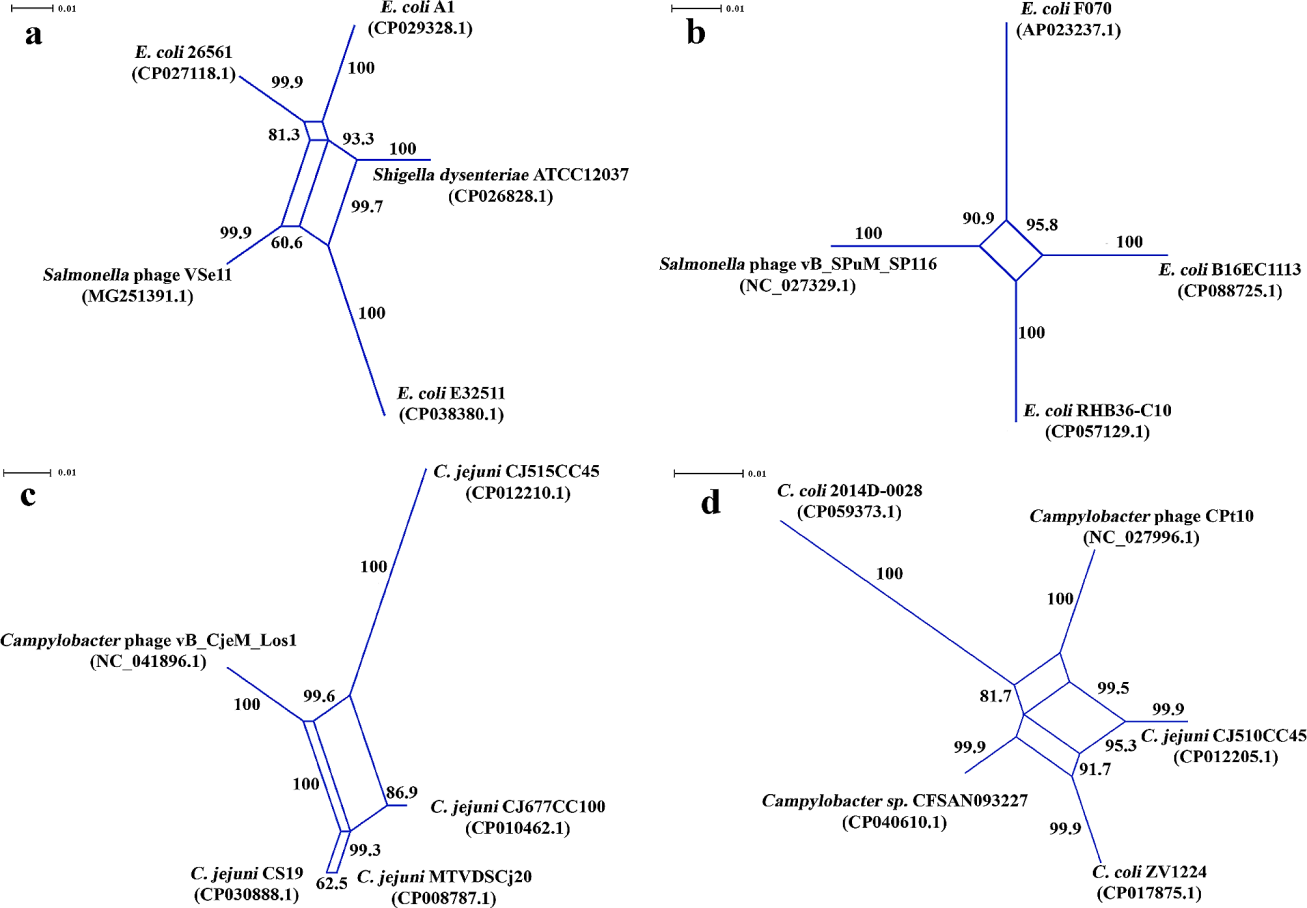
The SplitTree-generated parallelograms exhibiting conjointly the HGT events between the virulent *Salmonella* phages and some *E. coli* strains, and between the *Campylobacter* phages and *C. jejuni* versus *C. coli* strains. The HGT events identified between: (**a**) *Salmonella* phage VSe11 (MG251391.1) and *E. coli* strains, when analyzing the phage 544-bp chromosomal region involved in encoding for the hypothetical protein (GenBank protein ID: AUE22344.1) (Fit: 99.087); (**b**) *Salmonella* phage vB_SPuM_SP116 (NC_027329.1) and *E. coli* strains, when analyzing the phage 542-bp chromosomal region involved in encoding for the hypothetical protein (GenBank protein ID: YP_009146313.1) (Fit: 100); (**c**) *Campylobacter* phage vB_CjeM_Los1 (NC_041896.1/KX879627) and some *C. jejuni* strains, when analyzing the phage 469-bp chromosomal region involved in encoding for the hypothetical proteins (GenBank protein IDs: YP_009597191.1 and YP_009597192.1) (Fit: 99.969); (**d**) *Campylobacter* phage CPt10 (NC_027996.1/FN667789.1) and *C. jejuni*, and *C. coli* strains, when analyzing the phage 556-bp chromosomal region associated with the pseudogene (GenBank gene ID: 26,041,090) linked putatively to the protein motif PFAM (PF01555) (Fit: 91.433). In the splits graphs, the phage names appear according to their designations as presented in the NCBI GenBank database

In the genome collection of 15 *Salmonella* virulent phages examined, the SplitsTree analysis could determine the LGT signals across the 544-bp and 542-bp chromosomal regions of the *Salmonella* phages VSe11 (MG251391.1) and vB_SPuM_SP116 (NC_027329.1) respectively. In these phages, the above chromosomal regions were found to be involved in encoding for the hypothetical proteins. When examining the 544-bp region of the *Salmonella* phage VSe11 (MG251391.1) and its homologs, identified by BLAST across the chromosomes of multiple bacterial strains, the split decomposition analysis could generate three parallelograms exhibiting LGT events. As shown in Fig. 2a, these parallelograms were shared conjointly by the above phage, a single strain of *Shigella dysenteriae*, and three *E. coli* strains. A great majority of the bootstrap values, determined for the nodes of the parallelograms, were in a range of 93.3–100 supported by the very strong fit value being 99.087. In parallel, the SplitsTree-constructed single parallelogram with the highest fit and the robust bootstrap values (90.9–100), shown in Fig. 2b, exhibits the LGT events involving the *Salmonella* phage vB_SPuM_SP116 (NC_027329.1) and three *E. coli* strains. The DNA sequences of the LGT-targeted 544-bp and 542-bp homologous regions were reexamined by the Phi test, which resulted in *p*-values 0.003484 and 0.0191 respectively, revealing no evidence for convergent evolution in these genetic loci (Table 1).

The utility of the RDP4 algorithms allowed us to determine the recombination beginning and end breakpoints across the DNA sequence alignments of the 544-bp and 542-bp homologous regions (Table 3). Besides, as shown in Table 3, both *Salmonella* phages were determined to be the recombinant strains, while several *E. coli* strains (E32511, A1, B16EC1113, RHB36-C10, and F070) were inferred to be collectively their major or minor donors in the LGT events in these RDP4 analyses. Using RAST, the reannotation of the genomes of the *Salmonella* phages VSe11 (MG251391.1) and vB_SPuM_SP116 (NC_027329.1) showed that their targeted 544-bp and 542-bp genetic loci, playing a role in encoding for the hypothetical proteins, were of phage origin (Table S2); specifically, according to the RAST annotation, the coding sequences (CDS), for these proteins (GenBank protein IDs: AUE22344.1 and YP_009146313.1), represented the genes encoding for the phage proteins. However, it must be indicated that RAST failed in determining putative functions for these phage proteins.

**Table 3:** The results obtained from the RDP4 analyses exhibiting the recombination beginning and end breakpoints across the LGT-affected genetic loci, and the trajectories of the LGT events of these loci, involving the *Salmonella* phages and *E. coli* strains

Recombinant strain (GenBank acc. #)		Major donor (GenBank acc. #)		Minor donor (GenBank acc. #)	CDS, for a protein, within a phage genomic region examined (Coordinates in a phage genome [size in bps])	Recombination beginning and end breakpoints (99% Cl) *		*P*-value generated by the RDP4 algorithm	
*Salmonella* phage VSe11 (MG251391.1)		*E. coli* strain E32511 (CP038380.1)	*E. coli* strain A1 (CP029328)	Hypothetical protein (ID: AUE22344.1) CDS: <1.>544. (41445.41988 [544])	324 (454 − 440) – 484 (454 − 440)	RDP: GENECONV: BootScan: MaxChi: Chimaera: SiScan: 3Seq:		7.426 × 10^− 03^ 4.133 × 10^− 02^ 7.537 × 10^− 03^ 7.987 × 10^− 05^ 7.363 × 10^− 03^ 1.548 × 10^− 05^ 6.100 × 10^− 03^	
*Salmonella* phage vB_SPuM_SP116 (NC_027329.1)		*E. coli* strain B16EC1113 (CP088725.1)	*E. coli* strain F070 (AP023237.1)	Hypothetical protein (ID: YP_009146313.1) CDS: <1.>542. (45739.46280 [542])	402 (199 − 197) – 50 (199 − 197)	RDP: GENECONV: BootScan: MaxChi: Chimaera: SiScan: 3Seq:		9.653 × 10^− 03^ - 2.397 × 10^− 03^ 4.388 × 10^− 03^ 2.853 × 10^− 03^ - 5.797 × 10^− 03^	
*Salmonella* phage vB_SPuM_SP116 (NC_027329.1)		Unknown	*E. coli* strain RHB36-C10 (CP057129.1)	Hypothetical protein (ID: YP_009146313.1) CDS: <1.>542. (45739.46280 [542])	Undetermined (71[position 71 without gaps]) – Undetermined (225 [position 225 without gaps])	RDP: GENECONV: BootScan: MaxChi: Chimaera: SiScan: 3Seq:		- - - 2.707 × 10^− 02^ - 3.729 × 10^− 03^ 2.221 × 10^− 02^	

CDS - Coding Sequence

Phages names appear according to their designations presented in the NCBI GenBank database

Recombination beginning and end breakpoints (99% Cl) * - The recombination beginning and end breakpoints in the DNA alignment

The virulence life cycle inferences, made conceptually by PhageAI (Table S3) for the *Salmonella* phages VSe11 (MG251391.1) and vB_SPuM_SP116 (NC_027329.1) (prediction accuracy: 98.98% and 99.26% respectively), were in strong agreement with their actual life cycle. The actual life cycles of these phages could be determined previously under in vitro conditions [44, 45]. PHASTER could not identify prophage DNA sequences across the LGT-targeted chromosomal regions of the RDP4-determined *E. coli* donor strains (E32511, A1, B16EC1113, RHB36-C10, and F070) (Table S4).

In addition, the genetic recombination analyses could determine LGT events entailing the *Campylobacter* phages vB_CjeM_Los1 (NC_041896.1) and CPt10 (NC_027996.1) out of six virulent *Campylobacter* phages (Table S1), as well as some *C. jejuni* and/or *C. coli* strains. In particular, the split decomposition analysis of the BLAST-identified 469-bp homologous chromosomal regions of the *Campylobacter* phage vB_CjeM_Los1 (NC_041896.1) and four *C. jejuni* strains yielded three parallelograms (Fig. 2c). These parallelograms illuminated the fairly strong bootstrap estimates (93–100) for a majority of their nodes firmly supported by the very high fit (fit = 99.969). Similarly, three parallelograms (Fig. 2d) could be also generated by the split decomposition method when analyzing the 556-bp chromosomal region of the *Campylobacter* phage CPt10 (NC_027996.1) and its homologs determined by BLAST across the genomes of some *C. jejuni* and *C. coli* strains. These parallelograms were accompanied predominantly by the robust bootstrap estimates (in a range of 95.3–100) and a high fit value being 91.433. In the *Campylobacter* phage vB_CjeM_Los1 genome (NC_041896.1), the targeted 469-bp chromosomal region included the genetic loci of the genes encoding for two hypothetical proteins (GenBank protein IDs: YP_009597191.1 and YP_009597192.1). However, the 556-bp chromosomal region of the *Campylobacter* phage CPt10 (NC_027996.1) could be found to be a part of the pseudogene (GenBank gene ID: 26,041,090) partially linked to the protein motif PFAM (PF01555). *P*-values 0.04491 and 2.836E-12, computed by the Phi test for the 469-bp and 556-bp homologous chromosomal regions of these phage and bacterial strains respectively (Table 1), served as additional evidence supporting strongly the LGT-associated inferences obtained from the split decomposition analyses (Fig. 2c-d).

Using RDP4, we could determine the recombination beginning and end breakpoints identified across the DNA alignments of the above 469-bp and 556-bp homologous chromosomal regions of these organisms (Table 4). As shown, we could also determine the trajectories of the RDP4-identified multiple LGT events involving the above viral and bacterial chromosomal homologs: the *Campylobacter* phage vB_CjeM_Los1 (NC_041896.1) was determined to be the recombinant organism of the internal loci of the 469-bp chromosomal region, recombining with two *C. jejuni* strains (CP010462.1 and CP012210.1); these *C. jejuni* strains, in the above independent putative LGT events, could be found to have switched their roles of the major versus minor donors; in contrast, in a single LGT event, the *Campylobacter* phage CPt10 (NC_027996.1) was determined to be the minor donor of the targeted locus of the 556-bp chromosomal region for the recombinant *C. jejuni* strain CJ510CC45 (CP012205.1); however, this phage appeared to be the recombinant strain in the putative LGT event involving the *C. coli* strain 2014D-0028 (CP059373.1) as the major donor of the second locus of the same chromosomal region.

**Table 4:** The results obtained from the RDP4 analyses exhibiting the recombination beginning and end breakpoints across the LGT-affected genetic loci, and the trajectories of the LGT events of these loci, involving the *Campylobacter* phages and the *C. jejuni* strains

Recombinant strain (GenBank acc. #)		Major donor (GenBank acc. #)		Minor donor (GenBank acc. #)		CDS, for a protein, within a phage genomic region examined (Coordinates in a phage genome [size in bps])		Recombination beginning and end breakpoints (99% Cl) *	*P*-value generated by the RDP4 algorithm			
*Campylobacter* phage vB_CjeM_Los1 (NC_041896.1)	*C. jejuni* strain CJ677CC100 (CP010462.1)		*C. jejuni* strain CJ515CC45 (CP012210.1)		Hypothetical protein (ID: YP_009597191.1) CDS: <1.462. Hypothetical protein (ID: YP_009597192.1) CDS: 459.>469. (67472.67940 [469])		Undetermined (38–451) – Undetermined (38–451)			RDP: GENECONV BootScan: MaxChi: Chimaera: SiScan: 3Seq:	- - - 4.911 × 10^− 02^ - - 7.968 × 10^− 03^	
*Campylobacter* phage vB_CjeM_Los1 (NC_041896.1)	*C. jejuni* strain CJ515CC45 (CP012210.1)		*C. jejuni* strain CJ677CC100 (CP010462.1)		Hypothetical protein (ID: YP_009597191.1) CDS: <1.462. (67472.67940 [469])		106 (171 − 166) – 318 (171 − 166)			RDP: GENECONV: BootScan: MaxChi: Chimaera: SiScan: 3Seq:	- 8.752 × 10^− 03^ 5.660 × 10^− 04^ 3.728 × 10^− 05^ 1.307 × 10^− 03^ - 3.334 × 10^− 02^	
*C. jejuni* strain CJ510CC45 (CP012205.1)	*C. coli* strain ZV1224 (CP017875.1)		*Campylobacter* phage CPt10 (NC_027996.1)		PSEUDO (Gene ID: 26,041,090): <1.>556. (8819.9374 [556])		548 (332 − 29) – 56 (42–329)			RDP: GENECONV BootScan: MaxChi: Chimaera: SiScan: 3Seq:	- 1.783 × 10^− 02^ 2.020 × 10^− 03^ 2.780 × 10^− 03^ 3.578 × 10^− 04^ 1.631 × 10^− 04^ 1.342 × 10^− 04^	
*Campylobacter* phage CPt10 (NC_027996.1)	*C. coli* strain 2014D-0028 (CP059373.1)		*Campylobacter* sp. CFSAN093227 (CP040610.1)		PSEUDO (Gene ID: 26,041,090): <1.>556. (8819.9374 [556])		56 (534 − 359) – 262 (Undetermined)			RDP: GENECONV BootScan: MaxChi: Chimaera: SiScan: 3Seq:	- - - 5.911 × 10^− 03^ - 2.854 × 10^− 03^ -	

CDS - Coding Sequence

Phages names appear according to their designations presented in the NCBI GenBank database

Recombination beginning and end breakpoints (99% Cl) * - The beginning and end breakpoints in the DNA alignment

RAST failed in the annotation of the genes encoding for the above hypothetical proteins (GenBank protein IDs: YP_009597191.1 and YP_009597192.1) when analyzing the *Campylobacter* phage vB_CjeM_Los1 genome (NC_041896.1). However, RAST identified two hypothetical protein-CDSs, and the Type III restriction-modification system methylation subunit-encoding gene across the 556-bp chromosomal region of the *Campylobacter* phage CPt10 (NC_027996.1) (Table S2).

The results obtained from the phage classification analysis with PhageAI, inferring a temperate life cycle (with the prediction accuracy of 88.82% and 85.7% respectively) (Table S3), were in disagreement with the virulent life cycle reported previously for the *Campylobacter* phages vB_CjeM_Los1 [46] and CPt10 [47]. However, the BLAST analysis showed that the genome of the *Campylobacter* phage vB_CjeM_Los1 (NC_041896.1) was most similar (DNA identity = 98.19%; query coverage = 95%; E = 0.0) to that of the *Campylobacter* virulent phage CP30A (NC_018861.1) [48, 49] from the same *Fletchervirus* genus in the NCBI GenBank database. Similarly, the genome of the *Campylobacter* phage CPt10 (NC_027996.1) was most similar to that of the *Campylobacter* lytic phage CP20 (MK408758.1) [50] from the same *Firehammervirus* genus, both sharing 99.85% of DNA identity (query coverage = 99; E = 0.0).

Having analyzed the genomes of all the recombined *Campylobacter* strains, PHASTER could identify the incomplete prophage DNA sequences overlapping the above 469-bp chromosomal region in the genome of the *C. jejuni* strain (CP012210.1) (Table S4). As reported above, this *C. jejuni* strain was determined to have served as both the major donor and the minor donor for the recombinant *Campylobacter* phage vB_CjeM_Los1 in two putative independent LGT events determined by RDP4. The *Salmonella* phage vB_SosS_Oslo (NC_018279.1) was inferred by PHASTER to be most common to the above incomplete prophage identified in the *C. jejuni* strain genome (CP012210.1) (Table S4). In contrast, PHASTER could not identify prophage-associated DNA sequences across the 556-bp chromosomal homologous regions of the *C. jejuni* and *C. coli* recombining strains determined in the Splits decomposition and RDP4 analyses.

### Genetic recombination analyses of *Staphylococcus* phages

**Fig. 3:**
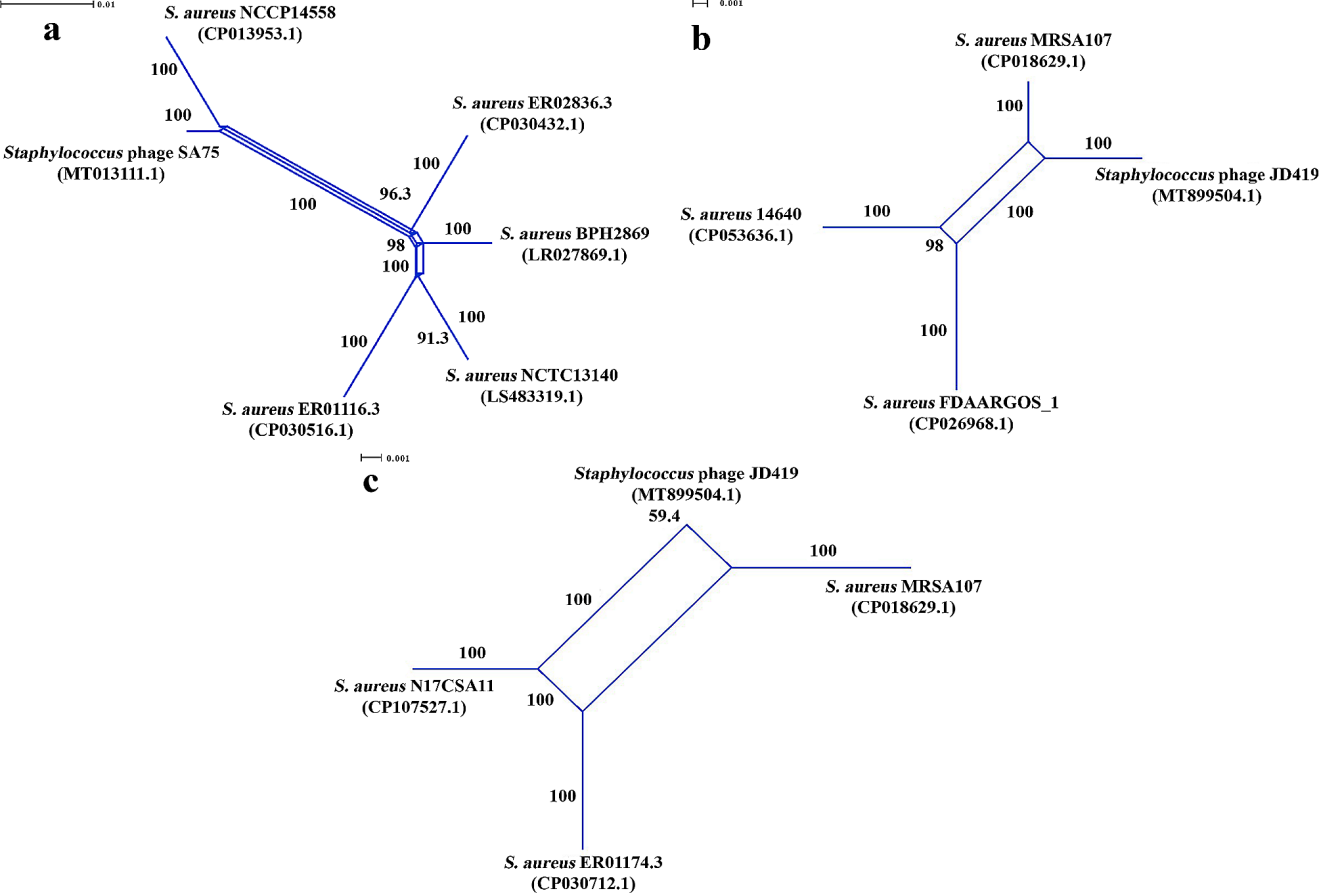
The SplitTree-generated parallelograms exhibiting conjointly the HGT events between the virulent *Staphylococcus* phages and *S. aureus* strains. The HGT events identified between: (**a**) *Staphylococcus* phage SA75 (MT013111.1) and some *S. aureus* strains, when analyzing the phage 27.05 kb chromosomal region containing a large number of genes encoding for terminase small and large subunits (GenBank protein IDs: QIA28729.1 and QIA28730.1 respectively), portal protein (GenBank protein ID: QIA28731.1), minor head protein (GenBank protein ID: QIA28732.1), capsid and scaffold protein (GenBank protein ID: QIA28734.1), major capsid protein (GenBank protein ID: QIA28735.1), tape measure protein (GenBank protein ID: QIA28744.1), putative distal tail protein (GenBank protein ID: QIA28745.1), putative tail associated lysin (GenBank protein ID: QIA28746.1), putative major teichoic acid biosynthesis protein C (GenBank protein ID: QIA28747.1), BppU family baseplate upper protein (GenBank protein ID: QIA28748.1), lysin, N-acetylmuramoyl-L-alanine amidase (GenBank protein ID: QIA28752.1), holin (GenBank protein ID: QIA28755.1), lysin (GenBank protein ID: QIA28756.1) and hypothetical proteins (GenBank protein IDs: QIA28733.1, QIA28736.1-QIA28743.1, QIA28749.1-QIA28751.1, QIA28753.1, QIA28754.1, and QIA28757.1) (Fit: 95.032); (**b-c**) *Staphylococcus* phage JD419 (GenBank protein ID: MT899504.1) and various S. aureus strains, when analyzing the phage 10.032 kb chromosomal region containing a large number of genes encoding for lysin, N-acetylmuramoyl-L-alanine amidase (GenBank protein ID: QOI66741.1), holin (GenBank protein ID: QOI66742.1), putative major teichoic acid biosynthesis protein C (GenBank protein ID: QOI66743.1), tail fiber (GenBank protein IDs: QOI66744.1 and QOI66745.1), tail length tape-measure protein (GenBank protein ID: QOI66746.1), and hypothetical proteins (GenBank protein IDs: QOI66719.1-QOI66723.1 ) (Fit: 100). In the splits graphs, the phage names appear according to their designations as presented in the NCBI GenBank database

In the SplitsTree and RDP4 analyses, examining a genome collection of the virulent phages including eight *Staphylococcus* phage (Table S1), we could identify also putative HGT events between two *Staphylococcus* phages and multiple *S. aureus* strains. In particular, the split decomposition analysis of the large chromosomal region (27.05 kb) of the *Staphylococcus* phage SA75 (MT013111.1) and its chromosomal homologous loci, identified by BLAST in the genomes of five *S. aureus* strains, could generate the splits graph encompassing five parallelograms (Fig. 3a). The nodes of the above parallelograms were highly supported by the bootstrap estimates being in a range of 96.3–100, being accompanied by the strong fit value 95.032. The Phi test-resulted *p*-value 0.0 (Table 1), obtained when examining the same subset of the DNA sequences, led to the no-convergent evolutionary scenario, thus, confirming firmly the above HGT events between the *Staphylococcus* phage SA75 and the *S. aureus* strains.

Also, the split decomposition analysis of the 10.032-kb homologous chromosomal regions, determined by BLAST, allowed us to identify the putative HGT events between the *Staphylococcus* phage JD419 (MT899504.1) and several other S. *aureus* strains. In particular, as shown in Fig. 3b-c, the SplitsTree-generated parallelograms included the nodes predominantly with the highest bootstrap and highest fit estimates. The Phi test could not reveal any evidence for convergent evolution across the 10.032-kb homologous chromosomal regions of the above organisms (*p* = 0.0) (Table 1).

Using the RDP4-embeeded recombination detection algorithms, we could determine multiple recombination beginning and end breakpoints across the 27.05-kb homologous regions of the above recombining organisms (Table 5 and S5). As shown, the above recombination beginning and end breakpoints exhibited at least fourteen putative LGT events, where the phage SA75 served as the recombinant strain or the minor donor of multiple genes and genetic loci for the *S. aureus* strains. The recombination beginning and end breakpoints, determined by the RDP4 analysis of the 10.032-kb homologous chromosomal regions, led to four putative LGT events involving the *Staphylococcus* phage JD419. In the RDP4 analysis, delineating these events, the *Staphylococcus* phage JD419 was determined to be the recombinant strain or the minor donor of various genes and gene loci for certain *S. aureus* strains (Table 6 and S5). The RDP4-produced *p*-statistics were strongly supportive of the above genetic recombination inferences. The recombined genetic loci, identified across the 27.05-kb and 10.032-kb chromosomal regions in these phages, were found to include a fairly large number of the important phage genes. Collectively, these genes encoded for lysin, holin, major teichoic acid biosynthesis protein C, tail length tape-measure protein, TMP, distal tail protein, tail fiber, and tail length tape-measure protein.

**Table 5:** The results obtained from the RDP4 analyses exhibiting the recombination beginning and end breakpoints across the LGT-affected genetic loci, and the trajectories of the LGT events of these loci, involving the *Staphylococcus* phage SA75 and the *S. aureus* strains

Recombinant strain (GenBank acc. #)			Major donor (GenBank acc. #)		Minor donor (GenBank acc. #)		CDS, for a protein, within a phage genomic region examined (Coordinates in a phage genome [size in bps])	Recombination beginning and end breakpoints (99% Cl) *		*P*-value generated by the RDP4 algorithm			
*Staphylococcus* phage SA75 (MT013111.1)	*S. aureus* strain NCCP14558 (CP013953.1)		*S. aureus* strain BPH2869 (LR027869.1)		Tape measure protein (ID: QIA28744.1) CDS: 9415.12879. Putative distal tail protein (ID: QIA28745.1) CDS: 12892.13839. Putative tail associated lysin (ID: QIA28746.1) CDS: 13848.15749. (1.27050 [27,050])			10,636 (10,404–10,736) – 14,387 (14,337–14,446)		RDP: GENECONV BootScan: MaxChi: Chimaera: SiScan: 3Seq:	6.446 × 10^− 65^ 1.225 × 10^− 48^ 2.050 × 10^− 31^ 6.039 × 10^− 28^ 1.380 × 10^− 30^ 7.734 × 10^− 16^ 1.243 × 10^− 14^		
* S. aureus* strain ER01116.3 (CP030516.1)	*S. aureus* strain NCTC13140 (LS483319.1)		*Staphylococcus* phage SA75 (MT013111.1)		Lysin, N-acetylmuramoyl-L-alanine amidase (ID: QIA28752.1) CDS: 20528.22426. Hypothetical protein (ID: QIA28753.1) CDS: 22439.23611. (1.27050 [27,050])			22,003 (21,332–22,023) – 22,760 (22,172 − 2234)		RDP: GENECONV BootScan: MaxChi: Chimaera: SiScan: 3Seq:	3.903 × 10^− 15^ 1.791 × 10^− 16^ 2.140 × 10^− 17^ 3.086 × 10^− 03^ 8.197 × 10^− 05^ 1.243 × 10^− 13^ 1.056 × 10^− 12^		
* S. aureus* strain BPH2869 (LR027869.1)	*S. aureus* strain ER01116.3 (CP030516.1)		*Staphylococcus* phage SA75 (MT013111.1)		BppU family baseplate upper protein (ID: QIA28748.1) CDS: 17674.19497. Hypothetical protein (ID: QIA28749.1) CDS: 19497.19874. Hypothetical protein (ID: QIA28750.1) CDS: 19875.20051. (1.27050 [27,050])			19,758 (19,716–19,774) – Undetermined (19,803–20,029)		RDP: GENECONV BootScan: MaxChi: Chimaera: SiScan: 3Seq:	1.177 × 10^− 09^ 1.480 × 10^− 08^ 2.619 × 10^− 10^ 3.271 × 10^− 04^ 7.150 × 10^− 06^ - 2.531 × 10^− 07^		
*S. aureus* strain NCTC13140 (LS483319.1)	*S. aureus* strain ER01116.3 (CP030516.1)		*Staphylococcus* phage SA75 (MT013111.1)		Hypothetical protein (ID: QIA28753.1) CDS: 22439.23611. (1.27050 [27,050])			Undetermined (22,961 [position 22,951 without gaps]) – 23,198 (undetermined)		RDP: GENECONV BootScan: MaxChi: Chimaera: SiScan: 3Seq:	- 3.650 × 10^− 02^ 8.368 × 10^− 01^ - - - -		
*Staphylococcus* phage SA75 (MT013111.1)	*S. aureus* strain NCCP14558 (CP013953.1)		Unknown		Capsid and scaffold protein (ID: QIA28734.1) CDS: 4698.5318. Major capsid protein, HK97 family (ID: QIA28735.1) CDS: 5332.6306. CDS of hypothetical proteins (IDs: QIA28736.1; QIA28737.1; QIA28738.1; QIA28739.1; QIA28740.1; QIA28741.1, QIA28742.1; QIA28743.1): 6328.6615; 6624.6956; 6953.7255; 7255.7602; 7614.7997; 8016.8597; 8659.9024; 9054.9398 respectively. Tape measure protein (ID: QIA28744.1) CDS: 9415.12879. Putative distal tail protein (QIA28745.1) CDS: 12892.13839. Putative tail associated lysin (QIA28746.1) CDS: 13848.15749. (1.27050 [27,050])			6603 (5230–6595) – Undetermined (6695–14,602)		RDP: GENECONV BootScan: MaxChi: Chimaera: SiScan: 3Seq:	- 3.682 × 10^− 05^ 2.623 × 10^− 03^ - - - 9.900 × 10^− 03^		
*S. aureus* strain ER02836.3 (CP030432.1)	*S. aureus* strain BPH2869 (LR027869.1)		*Staphylococcus* phage SA75 (MT013111.1)		CDS of Hypothetical proteins (IDs: QIA28749.1; QIA28750.1; QIA28751.1): 19497.19874; 19875.20051; 20092.20391 respectively. Lysin, N-acetylmuramoyl-L-alanine amidase (QIA28752.1) CDS: 20528.22426. CDS of hypothetical proteins (IDs: QIA28753.1; QIA28754.1): 22439.23611; 23617.24012 respectively. Holin (ID: QIA28755.1) CDS: 24069.24506. Lysin, N-acetylmuramoyl-L-alanine amidase (QIA28756.1) CDS: 24487.25932. (1.27050 [27,050])			Undetermined (19,690–25,342) – 25,094 (19,690–25,342)		RDP: GENECONV BootScan: MaxChi: Chimaera: SiScan: 3Seq:	1.845 × 10^− 02^ 1.540 × 10^− 02^ - - - - -		
*Staphylococcus* phage SA75 (MT013111.1)	Unknown		*S. aureus* strain NCTC13140 (LS483319.1)		Hypothetical protein (ID: QIA28751.1) CDS: 20092.20391. Lysin, N-acetylmuramoyl-L-alanine amidase (QIA28752.1) CDS: 20528.22426. (1.27050 [27,050])			Undetermined (20,195–21,216) – 21,134 (20,195–21,261)		RDP: GENECONV BootScan: MaxChi: Chimaera: SiScan: 3Seq:	- 2.445 × 10^− 02^ 3.135 × 10^− 02^ - - - -		

CDS - Coding Sequence

Phages names appear according to their designations presented in the NCBI GenBank database

Recombination beginning and end breakpoints (99% Cl) * - The recombination beginning and end breakpoints in the DNA alignment

**Table 6:** The results obtained from the RDP4 analyses exhibiting the recombination beginning and end breakpoints across the LGT-affected genetic loci, and the trajectories of the LGT events of these loci, involving the *Staphylococcus* phage JD419 and the *S. aureus* strains

Recombinant strain (GenBank acc. #)			Major donor (GenBank acc. #)		Minor donor (GenBank acc. #)		CDS, for a protein, within a phage genomic region examined (Coordinates in a phage genome [size in bps])	Recombination beginning and end breakpoints (99% Cl) *		*P*-value generated by the RDP4 algorithm			
*S. aureus* strain FDAARGOS_1 (CP026968.1)	*S. aureus* strain 14,640 (CP053636)		*Staphylococcus* phage JD419 (MT899504.1)		Lysin, N-acetylmuramoyl-L-alanine amidase (ID: QOI66741.1) CDS: Complement (759.2213). (30494.40525 [10,032])			122 (70–833) – Undetermined (1066–2170)		RDP: GENECONV BootScan: MaxChi: Chimaera: SiScan: 3Seq:	2.163 × 10^− 04^ 2.204 × 10^− 04^ 2.206 × 10^− 04^ 4.240 × 10^− 10^ 2.510 × 10^− 09^ 2.194 × 10^− 07^ 3.982 × 10^− 12^		
*Staphylococcus* phage JD419 (MT899504.1)	*S. aureus* strain MRSA107 (CP018629.1)		*S. aureus* strain N17CSA11 (CP107527.1)		CDS of hypothetical proteins (IDs: QOI66719.1; QOI66720.1; QOI66721.1; QOI66722.1; ): 2663.2962; 3008.3172; 3165.3554; 3554.5020. Putative major teichoic acid biosynthesis (ID: QOI66743.1) CDS: 5020.6930. Hypothetical protein (ID: QOI66723.1) CDS: 6946.7236. Tail fiber (ID: QOI66744.1) CDS: 7236.8819. Tail fiber (ID: QOI66745.1) CDS: 8828.9652. Tail length tape-measure protein (ID: QOI66746.1) CDS: 9652.>10,032. (30494.40525 [10,032])			3364 (2808–3620) – 10,026 (10016-13)		RDP: GENECONV BootScan: MaxChi: Chimaera: SiScan: 3Seq:	6.268 × 10^− 35^ 3.291 × 10^− 12^ 1.842 × 10^− 26^ 1.842 × 10^− 26^ 5.126 × 10^− 23^ 8.802 × 10^− 74^ -		
*S. aureus* strain N17CSA11 (CP107527_1)	*S. aureus* strain ER01174.3 (CP030712.1)		*Staphylococcus* phage JD419 (MT899504.1)		Lysin, N-acetylmuramoyl-L-alanine amidase (ID: QOI66741.1) CDS: 759.2213. (30494.40525 [10,032])			Undetermined (62–875) – Undetermined (1354–2156)		RDP: GENECONV BootScan: MaxChi: Chimaera: SiScan: 3Seq:	5.164 × 10^− 04^ 3.711 × 10^− 03^ 1.656 × 10^− 03^ 4.352 × 10^− 03^ 2.199 × 10^− 03^ 1.439 × 10^− 14^ 1.906 × 10^− 02^		

CDS - Coding Sequence

Phages names appear according to their designations presented in the NCBI GenBank database

Recombination beginning and end breakpoints (99% Cl) * - The recombination beginning and end breakpoints in the DNA alignment

In addition, RDP4 could also identify a large number of the recombined genetic loci involved in coding for the hypothetical proteins. Table S2 displays the results obtained from the RAST-applied reannotation of the recombined genes encoding for these hypothetical proteins in the *Staphylococcus* phages SA75 and JD419. As shown, among the recombining genes encoding for various hypothetical proteins, RAST could assign conceptually the functions only to three genes. Specifically, in the RAST analysis, the genes encoding for the hypothetical proteins under the GenBank IDs: QIA28753.1, QIA28736.1, and QIA28741.1, were determined to encode conceptually for the phage tail fiber, phage transcriptional terminator, and phage tail tube proteins respectively.

Specifically, with 99.25%- and 98.96%-prediction accuracy (Table S3), PhageAI inferred the temperate life cycle for the *Staphylococcus* phages SA75 (MT013111.1) and JD419 (MT899504.1). In our BLAST analysis, *Staphylococcus* phage SA75 (MT013111.1) was determined to be most closely related (DNA identity = 99.64%; query coverage = 77%; E = 0.0) to the *Staphylococcus* phage SA12 (NC_021801.1) from the same genus *Dubowvirus*. The *Staphylococcus* phage JD419 (MT899504.1) was found to be most closely related (DNA identity = 96.99%; query coverage = 88%; E = 0.0) to the uncharacterized *Staphylococcus* phage ECel-2020f (CP062442.1) from the same genus *Triavirus.* In our *in silico* analysis, PhageAI predicted (with 99.7% prediction accuracy) a temperate life cycle for the phage ECel-2020f (Table S3).

According to the PHASTER-generated results (Table S4), the genetic loci, acquired by the recombinant *Staphylococcus* phage SA75 in the above putative LGT events, were collectively associated with the chromosomal regions belonging to the prophages of the *S. aureus* strains NCCP14558 (CP013953.1) and BPH2869 (LR027869.1). Also, when serving as the minor donor in other LGT events, the *Staphylococcus* phage SA75 (MT013111.1) was determined to have interacted with the prophages of the *S. aureus* strains ER01116.3 (CP030516.1) and BPH2869 (LR027869.1), as well as with the incomplete prophages of the *S. aureus* strains NCTC13140 (LS483319.1) and ER02836.3 (CP030432.1) (Table S4). The genetic loci, exchanged putatively between the *Staphylococcus* phage JD419 (MT899504.1) and two *S. aureus* strains FDAARGOS_1 (CP026968.1) and N17CSA11 (CP107527_1) (Table 6), were determined to belong to the regions of intact prophages identified by PHASTER across the genomes of these strains (Table S4).

Among the *Staphylococcus* virulent phages examined in our *in silico* analyses, the 40,592-bp genome of the virulent phage SA97 (NC_029010.1) was determined to share the closest similarity with the chromosomal regions of various *S. aureus* strains. Specifically, the 32,000-bp genomic region of the *Staphylococcus* phage SA97 was found to be identical to a specific chromosomal region (across 10% query coverage; genome coordinates: 852807.884806) of the *S. aureus* strain ATCC 12,600 (CP035101.1). In the genome of this *S. aureus* strain, the above chromosomal region appeared to be a part of the intact prophage (coordinates: 833719.885549) identified by us using PHASTER (Table 4). The other remaining 8592-bp region of the phage SA97 genome was determined to share the closest DNA identity (99.73%) (across 100% query coverage) with one of the regions (coordinates: 840628.849141) of the same intact prophage of the *S. aureus* strain ATCC 12,600, as well as with the chromosomal regions of multiple other *S. aureus* strains in the GenBank database. PhageAI classified (with 99.15% prediction accuracy) the phage SA97 as a temperate phage.

## DISCUSSION

Various phage applications have been implemented, or are underway, across different clinical, agricultural, and other settings [51–53], targeting sometimes even human and animal distal intestines representing one the richest microbial habitats [54]. It can be thought that in microbially rich environments, especially in human and animal intestines, when being subjected to unprecedently high loads of virulent phages applied as part of therapeutic or food versus environmental safety measures, the opportunities for HGT-mediated phage-phage and phage-host interactions can increase enormously facilitating genetic variability of these organisms. Phage–host coevolutionary interactions, being heavily mediated by HGT [55], can influence the larger microbiomes [56] while modifying phage genome evolution, and even dictating sometimes the fate of their hosts [22]. In the last three decades, there has been a significant expansion of the phage armamentarium against the escalating threat of antimicrobial resistance. In this light, the gaining of deeper insights into the impact of LGT on the coevolutionary trajectories of industrial phages and their host networks has been highly desirable for advanced assessments of “One Health” risks that can be associated with phage-driven microbiome changes across various targeted industrial and other settings [22].

### LGT between *Escherichia* and *Listeria* virulent phages and their host prophages

The phenomenon of genetic exchange between *E. coli* virulent phages and their hosts remains largely unclear, although, it appears that both sides can act as donors and recipients of some genetic loci during their interactions. For example, the N4-like lytic phage EC1-UPM, infecting avian pathogenic *E. coli* O78:K80 [57], was suggested to be a major donor of the DNA primase gene for the *Enterococcus faecium prophage*, while the latter was found to be a donor of the tail protein-encoding gene locus for another *Escherichia* virulent phage, vB_EcoP_G7C in independent LGT events [22]. Here, the results of our study provide amplifying strong evidence for LGT events, which could involve certain *E. coli* prophages serving as putative donors of the phage tail TMP-encoding gene loci for the *E. coli* virulent phage vB_EcoM_DE7 proposed earlier as a biocontrol agent for treating foal diarrhea caused by *E. coli* [43]. The tail TMP is an important protein, because it forms a channel-like structure in a cytoplasm of phage-targeted hosts, allowing viral genome entry [58]. Thus, it is possible that, in certain instances, by involving the above phage genes, LGT can have some impact on the infective and/or replicative abilities of *E. coli* virulent phages and temperate phages of their shared hosts after completing their lysogenic life cycle.

Here, we also show, for the first time that, acting as the donors, *L monocytogenes* virulent phages could exchange gp55-encoding genetic loci with the *L. monocytogenes* prophages. Being a highly diverged member of the *σ*^70^ family, gp55 (the basal promoter specificity factor) represents the phage-encoded RNAP binding protein that contributes to the transcription activation of more than one-third of the phage genome [59]. It is noteworthy that the prophage transcriptional regulator (TR) genes are suggested to play some roles in regulating the host cell metabolism, contributing to a phage host survival and its environmental adaptation [60]. Hence, it is safe to assume that, when acting as a donor of certain TR gene loci, *L monocytogenes* virulent phages sometimes can have some indirect effect on the physiology of *L. monocytogenes* via specific LGT-mediated genomic alterations of its prophages. Besides, it is also strongly suggested that, *L. monocytogenes* virulent phages have the ability to acquire from *L. monocytogenes* prophages fragments of specific genes with unknown functions, and that some of these donor prophages can reside as a circular prophage in the phage host genome during such LGT events.

### LGT between *Salmonella* and *Campylobacter* virulent phages and prophages

Here, we provide first strong statistical evidence for LGT events between certain *Salmonella* virulent phages and *E. coli* prophages, involving genetic loci with unknown functions. In particular, it is shown that the *Salmonella* virulent phages vB_SPuM_SP116 and VSe11, which infect respectively various *Salmonella pullorum* versus *S. enterica* strains [44, 45], could serve as the recombinant organisms in these LGT events. It is important to note that the phage vB_SPuM_SP116 was reported to be a potential therapeutic candidate for the treatment of *S. Pullorum* infections [45]. Thus, similar to *E. coli* virulent phages, genomes of at least some *S. enterica* virulent phages could be affected by intergeneric recombination events inserting DNA fragments with new mutations across the hypothetical protein-encoding genes. The functions of these genes, and the nature of their genetic variations, occurring due to LGT, should be determined to understand whether such intergeneric recombination events can change the evolutionary trajectories and subsequently the fate of *S. enterica* recombinant virulent phages.

Our study also finds the first strong statistical evidence for the LGT events between certain *Campylobacter* virulent phages and prophages or the defective one carried by the *C. jejuni* and *C. coli* genomes. Interestingly, it is shown that the *C. jejuni* defective prophage, that acted as the donor for one of *C. jejuni* virulent phages, was genetically most closely related to the *Salmonella* temperate phage as determined by PHASTER. This finding allows us to suggest that some *C. jejuni* virulent phages, when interacting with their main host species, could acquire certain genetic loci from prophage elements representing the remnants of former *Salmonella* temperate phages integrated previously in the *C. jejuni* genome. Generally, we strongly suggest that *Campylobacter* virulent phages can act as both recombinants and donors at minimum for *C. jejuni* in LGT events, involving not only some phage genes, but also the genetic loci of bacterial origin belonging to the Type III restriction-modification (RM) system methylation subunit; specifically, here we show that the virulent phage CPt10 could serve as the recombinant organism and the donor of the genetic loci of the above subunit for the *C. jejuni* and *C. coli* strains in two independent LGT events. It is important to indicate that, the Type III RM systems belong to the defense machinery employed by a bacterial host against phage invasion [61]. This finding raises an important question as to whether the LGT-mediated phage-host coevolutionary interactions involving such genetic loci can promote the natural emergence of *Campylobacter* strains highly resistant to selected industrial *Campylobacter* virulent phages when applied e.g., as part of food safety strategies in poultry or other settings.

### HGT between *Staphylococcus* phages and prophages

It is thought that natural or engineered lytic variants of specific temperate phages hold promise for their effective therapeutic use [25, 62]. On this note, health and safety considerations have to be taken, knowing that the vast majority of temperate phages are involved in high gene content flux (HGCF) in contrast to virulent phages linked usually to a low gene content flux (LGCF) mode [21]. In general, phages easily regain as well as gain genetic mobility and new abilities by recombining with other phages and prophages [19, 63], whereby they can sometimes unprecedentedly alter surrounding native microbiomes [56]. Besides, HGT-induced phage-prophage interplays were found to alter not only the fitness and survival of their hosts under unfavorable conditions [4, 64, 65], but also the induction, propagation, and transmission of their prophages [66]. Thus, while the phage life cycle has been an important factor requiring extensive studies to possibly predict how industrial virulent phages can behave, significantly more research efforts need to be made to understand possible outcomes of the use of industrial temperate phages and their modified forms across different therapeutic, food safety and other settings.

Here, we demonstrate LGT events that could occur between the *S. aureus* phage SA75 and several *S. aureus* intact versus defective prophages. The above phage was proposed previously to be an alternative to the traditional antibiotics for treating *Staphylococcus* infections [23]. However, it is noteworthy that the genome of SA75, being most closely related to the *S. aureus* temperate phage SA12 [67], shows very strong patterns of the temperate phage life cycle as predicted by PhageAI in our *in silico* analyses. It is also shown that the above phage genome has been affected fairly significantly by a large number of the LGT events exhibiting homologous recombination. Homologous recombination events could affect, although to a relatively lesser degree, as well the genome of another *S. aureus* temperate phage, JD419 proposed earlier to be genetically modified for formulating it as a potentially effective therapeutic agent [24, 25]. Switching their roles from a donor to a recipient, the above *S. aureus* phages demonstrated the abilities allowing them to recombine with their host prophages fairly large clusters of the genes encoding for important phage proteins. Specifically, our analysis could determine the LGT events involving the endolysin-encoding genes, being in line with the previous findings, which had suggested that these genes can be naturally exchanged by homologous recombination between phages and prophages, contributing to their adaptation to new host environments [20]. Besides, HGT events, via the host prophages serving as donors, could also affect the holin-encoding genes in the above *Staphylococcus* temperate phages. It must be noted that in the lytic process, the holins act as the hydrophobic membrane proteins forming pores or lesions in a host cell membrane, allowing the transit of phage murein hydrolases to the periplasm [68]. It appears that the major teichoic acid biosynthesis protein C-encoding gene could be also shared by the above phages and their host prophages during LGT; this gene contributes to the adsorption of phage to its host cell [69]. In the *Staphylococcus* phage SA75, while being interacted with its host prophages, LGT could also affect the genes encoding for the phage transcriptional terminator protein (TTP), the capsid and scaffold protein, as well as the BppU family baseplate upper protein. Among these proteins, TTPs confer important broad functions linked e.g., to a modulation of the host RNA polymerase during transcription of the phage genomes [70], having an impact on different processes including, but not limited to, phage stress responses, its virulence, and amino acid transportation in its cell [60]. As shown, a plethora of genes with unknown functions could be also shared between the above temperate phages and the intact versus defective prophages of their hosts. Importantly, these genes were only a part of the recombined large chromosomal regions including also the tail-associated genetic loci, e.g., such as the TMP-encoding gene that dictates the tail length and facilitates DNA transit to the cell cytoplasm during infection [71]. The TMP gene seems to have been a frequent target of LGT in *S. aureus* phages. Moreover, similar to some other phage genes, described above, it has been involved in intergeneric recombination events [22]. Our study finds that due to its interactions with certain *S. aureus* prophages, the genomic regions of the temperate phage SA75 that encompassed the genes encoding for the tail fiber, distal tail, and tail tube proteins could also be affected by LGT. It is noteworthy that the tail fibers (or spikes), located at a distal end of the tail, serve as a core environment for the adsorption devise assembly of long-phage tails [72], and are believed to interact with polysaccharidic receptors located on the cell surfaces of phage hosts [73]; interestingly, the previous findings [22, 74] suggest that the phage tail fiber protein-encoding genes could be entailed even in intergeneric recombination; more importantly, the exchange of these genes was reported to mediate acquisition of diverse host range determinants allowing phages to cross their host species boundaries and infect taxonomically distant bacteria [75]; besides, it is assumed that the LGT-promoted extensive variability of receptor specificity will increase even more, and become almost limitless in phage populations [76]. As for the tail tube protein, it is a main elementary unit, whereby a phage injects its genome into a host cell [77], while playing its role also in signal transduction from the distal end of the tail to the capsid [78].

The above-detected LGT events serve as amplifying evidence for the exchange of the large genomic regions observed previously in various phages [79, 80]. The same phenomenon may appear to be explanatory for the genome organization of the *S. aureus* phage SA97 examined in our study; earlier, it was classified to be a virulent phage, and was proposed to be a promising candidate for controlling *S. aureus* infections [26]; in addition, it was also reported that SA97 could not be affected by LGT, even possessing the genes encoding for a lysogeny module [26]. Here, we show, in contrast, that the largest section of this phage chromosome (32,000-bp in size) is identical exclusively to the prophage region of the single *S. aureus* strain (ATCC 12,600), while the rest of its DNA material is genetically most closely related to the respective genomic regions of both the same prophage and multiple other *S. aureus* prophages. Hence, coupled with the PhageAI- and PHASTER-generated inferences obtained in our *in silico* analysis, these findings are alternatively suggestive of the temperate nature of the *S. aureus* phage SA97.

## Conclusions

We strongly suggest that specific *L. monocytogenes* virulent phages could serve at least as the donors of certain loci of the gene encoding for the basal promoter specificity factor for *L. monocytogenes*, while some of these phages could be also the recipients of multiple genetic loci with unknown functions when interacting with the above host species. Intergeneric recombination is suggested to take place, targeting certain genes of unknown functions, also in specific *E. coli* and *S. enterica* virulent phages during their interaction with their host prophages. Exhibiting intraspecies recombination of the genetic loci and/or genes with unknown functions, the Phage-host interaction-mediated LGT events could affect as well the genomes of the *C. jejuni* and *S. aureus* virulent and temperate phages. It is also suggested that the above *S. aureus* temperate phages could acquire from, or donate to, their host (*S. aureus*), via a single LGT event, simultaneously some of these and other genes involved in the synthesis of the capsid and scaffold proteins, phage tail proteins, holins, and TTPs. In the *S. aureus* temperate phages and prophages, LGT could affect significantly larger chromosomal regions as compared to those observed across the genomes of the *E. coli*, *S. enterica*, *L. monocytogenes*, and *C. jejuni* virulent phages and their hosts. Some of the above-detected LGT events may exhibit the genetic interactions between a virulent phage and a temperate phage prior to a switching of the latter to a prophage state.

### Electronic supplementary material

Below is the link to the electronic supplementary material.

Supplementary Material 1

## Data Availability

The datasets analyzed during the current study are publicly available in the National Center for Biotechnology Information repository (https://www.ncbi.nlm.nih.gov/) under the following GenBank accession numbers: OL825705.1; CP088725.1; CP010206; CP021325.1; CP007689.1; NC_024360.1; NC_042048.1; NC_047871.1; LR134403.1; MG251391.1; CP038380.1; CP029328.1; NC_027329.1; AP023237.1; CP057129.1; NC_041896.1; CP010462.1; CP012210.1; CP012205.1; CP017875.1; NC_027996.1; CP059373.1; CP040610.1; MT013111.1; CP013953.1; LR027869.1; CP030516.1; LS483319.1; CP030432.1; CP026968.1; CP053636.1; MT899504.1; CP018629.1; CP107527.1; CP030712.1; CP013722.1; LR698978.1; CP027118.1; CP026828.1; CP030888.1; CP008787.1.

## Abbreviations

LGT: Lateral genetic transfer
IBD: inflammatory bowel disease
BLAST: Basic Local Alignment Search Tool
PHASTER: PHAge Search Tool-Enhanced Release
TMP: Phage tail tape measure protein
CDS: Coding sequences
TR: Transcriptional regulator
RM: Restriction-modification
HGCF: high gene content flux
LGCF: Low gene content flux
TTP: Transcriptional terminator protein
