# Supplementary material for: Genetic recombination-mediated evolutionary interactions between phages of potential industrial importance and prophages of their hosts within or across the domains of Escherichia, Listeria, Salmonella, Campylobacter, and Staphylococcus
Source: BMC Microbiol. 2024 May 4;24:155. doi: 10.1186/s12866-024-03312-6 (PMC11069274; doi:10.1186/s12866-024-03312-6)
Supplement: Supplementary file 1 — Supplementary Material 1 [file 12866_2024_3312_MOESM1_ESM.docx]

**SUPPLEMENTARY INFORMATION**

**Genetic Recombination-Mediated Evolutionary Interactions between Phages of Potential Industrial Importance and Prophages of Their Hosts within or across the Domains of *Escherichia, Listeria*, *Salmonella, Campylobacter*, and *Staphylococcus***

Saba Kobakhidze ^1, 2^, Stylianos Koulouris ^3^, Nata Kakabadze ^1^, Mamuka Kotetishvili ^1, 4*^.

^1^G. Natadze Scientific-Research Institute of Sanitary, Hygiene and Medical Ecology, 78 D. Uznadze St. 0102, Tbilisi, Georgia.

^2^Faculty of Medicine, Iv. Javakhishvili Tbilisi State University, 1 Ilia Chavchavadze Ave. 0179, Tbilisi, Georgia.

^3^European Commission, Directorate General for Health and Food Safety (DG-SANTE), 1049 Bruxelles/Brussel, Belgium.

^4^Scientific Research Institute, School of Science and Technology, the University of Georgia, 77a M. Kostava St. Tbilisi, 0171, Georgia.

***Corresponding author:** Mamuka Kotetishvili

**E-mail:** [m.kotetishvili@hygiene.ge](mailto:m.kotetishvili@hygiene.ge)

ORCID #: 0000-0002-3882-0878

**Table S1.** The GenBank Accession numbers for the phage genomes examined in the study

| **No.** | **Phage designation** | **GenBank accession # for a phage genome** |
| --- | --- | --- |
| 1. | *Escherichia* phage phiC120 | KY703222.1 |
| 2. | *Escherichia* phage EcS1 | NC_055721.1 |
| 3. | *Escherichia* phage vB_EcoM_DE7 | OL825705.1 |
| 4. | *Escherichia coli* bacteriophage rv5 | DQ832317.1 |
| 5. | *Escherichia* phage vB_EcoS_Uz-1 | OP312987.1 |
| 6. | *Escherichia* phage BRET | MK165087.1 |
| 7. | *Escherichia* phage 590B | MW722821.1 |
| 8. | *Escherichia* phage vB_EcoM_SCS57 | OM960734.1 |
| 9. | *Escherichia* phage vB_EcoS_SCS44 | ON001686.1 |
| 10. | *Escherichia* phage vB_EcoS_SCS31 | ON081052.1 |
| 11. | *Escherichia* phage vB_EcoM_SCS4 | ON506924.1 |
| 12. | *Escherichia* phage vB_EcoS_SCS92 | ON548432.1 |
| 13. | *Escherichia* phage vB_EcoP_FFK3 | ON548433.1 |
| 14. | *Escherichia* phage Ro45lw | MK301532.1 |
| 15. | *Escherichia* phage vb_EcoM_bov9_1 | MT884006.2 |
| 16. | *Escherichia* phage vb_EcoM_bov10K1 | MT884007.2 |
| 17. | *Escherichia* phage vb_EcoM_bov10K2 | MT884008.2 |
| 18. | *Escherichia* phage vb_EcoM_bov11CS3 | MT884009.2 |
| 19. | *Escherichia* phage vb_EcoM_bov22_2 | MT884010.2 |
| 20. | *Escherichia* phage vb_EcoS_bov11C2 | MT884012.2 |
| 21. | *Escherichia* phage vb_EcoS_bov16_1 | MT884013.2 |
| 22. | *Escherichia* phage vB_EcoS_bov15_1 | MT951623.2 |
| 23. | *Escherichia* phage vB_EcoM_C2-3 | OK076929.1 |
| 24. | *Escherichia* phage ECBP2 | JX415536.1 |
| 25. | *Escherichia* phage vB_EcoM_ECO1230-10 | NC_027995.1 |
| 26. | *Escherichia* phage T4 | AF158101.6 |
| 27. | Escherichia phage ST31 | NC_047829.1 |
| 28. | *Escherichia* phage ST20 | MF153391.1 |
| 29. | *Escherichia* phage phAPEC8 | JX561091.1 |
| 30. | Enterobacteria phage AR1 DNA | AP011113.1 |
| 31. | Enterobacteria phage NJ01 | JX867715.1 |
| 32. | *Salmonella* phage vB_SenTO17 | MT012729.1 |
| 33. | *Salmonella* phage wksl3 | JX202565.1 |
| 34. | *Salmonella* phage ZCSE2 | MK673511.1 |
| 35. | *Salmonella* phage vB_SpuP_Spp16 | NC_047941.1 |
| 36. | *Salmonella* phage IME207 | KX523699.2 |
| 37. | *Salmonella* virus VSe11 | MG251391.1 |
| 38. | *Salmonella* phage vB_SalM-LPST153 | MK907285.1 |
| 39. | *Salmonella* phage vB_SPuM_SP116 | NC_027329.1 |
| 40. | *Salmonella* phage SE2 | JQ007353.1 |
| 41. | *Salmonella* phage SS3e | AY730274.2 |
| 42. | *Salmonella* phage PMBT28 | MG641885.1 |
| 43. | *Salmonella* phage NBSal007 | MT677934.1 |
| 44. | *Salmonella* phage NBSal006 | MT677933.1 |
| 45. | *Salmonella* phage PRF-SP1 | MZ923531.1 |
| 46. | *Salmonella* phage vB_SenM_PA13076 | MF740800.1 |
| 47. | *Listeria phage* P35 | DQ003641.1 |
| 48. | *Listeria* phage P40 | EU855793.1 |
| 49. | *Listeria* phage List-36 | NC_024364.1 |
| 50. | *Listeria* phage LMTA-94 | NC_047872.1 |
| 51. | *Listeria* phage P70 | NC_018831.1 |
| 52. | *Listeria* phage LMSP-25 | NC_024360.1 |
| 53. | *Listeria* phage LMTA-34 | NC_042048.1 |
| 54. | *Listeria* phage LMTA-57 | NC_047871.1 |
| 55. | *Campylobacter* phage vB_CjeM_Los1 | NC_041896.1 |
| 56. | *Campylobacter* phage CP220 | NC_027997.1 |
| 57. | *Campylobacter* phage CPt10 | NC_027996.1 |
| 58. | *Campylobacter* phage vB_CjeM_Los1 | KX879627.1 |
| 59. | *Campylobacter* phage CP220 | FN667788.1 |
| 60. | *Campylobacter* phage vB_CcoM-IBB_35 | HM246720.1 |
| 61. | *Staphylococcus* phage JD007 | NC_019726.1 |
| 62. | *Staphylococcus* phage JD419 | MT899504.1 |
| 63. | *Staphylococcus* phage SA97 | NC_029010.1 |
| 64. | *Staphylococcus* phage vBSM-A1 | MK584893.1 |
| 65. | *Staphylococcus* phage SA75 | MT013111.1 |
| 66. | *Staphylococcus* phage Maine | MN045228.1 |
| 67. | *Staphylococcus* phage pSa-3 | KY581279.1 |
| 68. | *Staphylococcus* phage YMC/09/04/R1988 | NC_022758.1 |
| 69. | *Enterococcus* phage EFAP-1 | FJ792813.1 |
| 70. | *Lactobacillus* phage Lb338-1 | FJ822135.1 |
| 71. | *Citrobacter* phage phiCFP-1 | KP313531.1 |
| 72. | *Pseudomonas* phage vB_PaeP_PaCe | ON376263.1 |
| 73. | *Pseudomonas* phage vB_PsyM_KIL1 | KU130126.1 |
| 74. | *Pseudomonas* phage vB_PsyM_KIL2 | KU130127.1 |
| 75. | *Pseudomonas* phage vB_PsyM_KIL3 | KU130128.1 |
| 76. | *Pseudomonas* phage vB_PsyM_KIL3b | KU130131.1 |
| 77. | *Pseudomonas* phage PPpW-3 DNA | AB775548.1 |
| 78. | *Pseudomonas* phage PPpW-4 DNA | AB775549.1 |
| 79. | *Pseudomonas* phage PN09 | MW175491.1 |
| 80. | *Pseudomonas* phage vB_PaeM_SCUT-S1 | MK340760.1 |
| 81. | *Pseudomonas* phage vB_PaeM_SCUT-S2 | MK340761.1 |
| 82. | *Pseudomonas* phage PAXYB1 | KY618819.1 |
| 83. | *Pseudomonas* phage PB1 | NC_011810.1 |
| 84. | *Pseudomonas* phage vB_PaeP_PPA-ABTNL | KM067278.1 |
| 85. | *Pseudomonas* phage phiPSA2 | NC_024362.1 |
| 86. | *Pseudomonas* phage phikF77 | NC_012418.1 |
| 87. | *Pseudomonas* phage PMBT14 | NC_048687.1 |
| 88. | *Pseudomonas* phage SaPL | MH973725.1 |
| 89. | *Pseudomonas* phage PMBT14 | MG596800.2 |
| 90. | *Pseudomonas* phage PMBT3 | MG596799.1 |
| 91. | *Xanthomonas* phage Samson | MN062187.1 |
| 92. | *Aeromonas* phage 25AhydR2PP | MH179473.2 |
| 93. | *Aeromonas* phage 50AhydR13PP | MH179476.1 |
| 94. | *Aeromonas* phage phiAS5 | NC_014636.1 |
| 95. | *Aeromonas* phage MJG | MK455769.1 |
| 96. | *Acinetobacter* phage vB_AbaS_Loki | LN890663.1 |
| 97. | *Vibrio* phage SHOU24 | NC_023569.1 |
| 98. | *Vibrio* phage VAP7 | NC_048765.1 |
| 99. | *Vibrio* phage CHOED | NC_023863.2 |
| 100. | *Vibrio* phage PVA1 | NC_023605.1 |
| 101. | *Vibrio* phage VpKK5 | NC_026610.2 |

Phages names appear according to their designations presented in the NCBI GenBank database.

**Table S2.** The RAST annotation of the LGT-affected phage genes encoding for hypothetical proteins

| **Phage designation**  **(GenBank acc. #) and taxonomic status** | **Phage hypothetical protein GenBank ID** | **Phage genome coordinates for a protein CDS in GenBank** | **A RAST-predicted protein for a gene** | **Phage genome coordinates of a CDS for a RAST-predicted protein** |
| --- | --- | --- | --- | --- |
| *Escherichia* phage vB_EcoM_DE7  (OL825705.1)  (*Caudoviricetes*; *Ounavirinae*; *Felixounavirus*) | [UKH49269.1](https://www.ncbi.nlm.nih.gov/protein/2189060779) | 30355..31152 | Phage tail tape measure protein | 30355..31152 |
| *Listeria* phage LMSP-25  (NC_024360.1)  (*Caudoviricetes; Herelleviridae Jasinkavirinae*; *Pecentumvirus*) | [YP_009043029.1](https://www.ncbi.nlm.nih.gov/protein/658307331) | 21673..21882 | Protein gp55  [Bacteriophage A118] | 21673..21882 |
|  | [YP_009043030.1](https://www.ncbi.nlm.nih.gov/protein/658307332) | 21879..22283 | Phage protein | 21879..22283 |
| *Listeria* phage LMTA-34  (NC_042048.1)  (*Caudoviricetes; Herelleviridae Jasinkavirinae; Pecentumvirus*) | [YP_009616147.1](https://www.ncbi.nlm.nih.gov/protein/1631938429) | 21673..21882 | Protein gp55  [Bacteriophage A118] | 21673..21882 |
|  | [YP_009616148.1](https://www.ncbi.nlm.nih.gov/protein/1631938430) | 21879..22283 | Phage protein | 21879..22283 |
| *Listeria* phage LMTA-57  (NC_047871.1)  (*Caudoviricetes; Herelleviridae Jasinkavirinae; Pecentumvirus*) | [YP_009793497.1](https://www.ncbi.nlm.nih.gov/protein/1842006290) | Complement  (135433..135837) | Phage protein | Complement  (135433..135837) |
|  |  |  |  |  |
| *Salmonella* phage VSe11 (MG251391.1)  (*Caudoviricetes; Ounavirinae*; *Felixounavirus*) | [AUE22344.1](https://www.ncbi.nlm.nih.gov/protein/1308004411) | 41424..42221 | Phage protein | 41424..42221 |
|  |  |  |  |  |
| *Salmonella* phage vB_SPuM_SP116  (NC_027329.1)  *Caudoviricetes; Ounavirinae; Felixounavirus* | [YP_009146313.1](https://www.ncbi.nlm.nih.gov/protein/849248427) | 45718..46515 | Phage protein | 45718..46515 |
|  |  |  |  |  |
| *Campylobacter* phage vB_CjeM_Los1  (NC_041896.1) | [YP_009597191.1](https://www.ncbi.nlm.nih.gov/protein/1631919294) | 67286..67933 | Hypothetical protein | 67286..67933 |
| (*Caudoviricetes;Eucampyvirinae*; *Fletchervirus*) | [YP_009597192.1](https://www.ncbi.nlm.nih.gov/protein/1631919295) | 67930..68274 | Hypothetical protein | 67930..68274 |
|  |  |  |  |  |
| *Campylobacter* phage CPt10  (NC_027996.1)  (*Caudoviricetes*; *Eucampyvirinae*; *Firehammervirus*) | PSEUDO (Gene ID: [26041090](https://www.ncbi.nlm.nih.gov/gene/26041090)) | 7773..9384 | Hypothetical protein; hypothetical protein; Type III restriction-modification system methylation subunit | 6700..7776; 7806..9023;  9088..9384. |
|  |  |  |  |  |
| *Staphylococcus* phage SA75 (MT013111.1)  (*Caudoviricetes; Azeredovirinae*; *Dubowvirus*) | [QIA28753.1](https://www.ncbi.nlm.nih.gov/protein/1808644108) | 22439..23611 | Phage tail fiber | 22439..23611 |
|  | [QIA28749.1](https://www.ncbi.nlm.nih.gov/protein/1808644104) | 19497..19874 | Bacteriophage hypothetical protein homolog | 19497..19874 |
|  | [QIA28750.1](https://www.ncbi.nlm.nih.gov/protein/1808644105) | 19875..20051 | Bacteriophage hypothetical protein homolog | 19875..20051 |
|  | [QIA28751.1](https://www.ncbi.nlm.nih.gov/protein/1808644106) | 20092..20391 | Hypothetical protein, SLT orf99 homolog [SA bacteriophages 11, Mu50B | 20092..20391 |
|  |  |  |  |  |
|  | [QIA28736.1](https://www.ncbi.nlm.nih.gov/protein/1808644091) | 6328..6615 | Phage transcriptional terminator | 6328..6615 |
|  | [QIA28737.1](https://www.ncbi.nlm.nih.gov/protein/1808644092) | 6624..6956 | phi 11 orf36 homolog [SA bacteriophages 11, Mu50B] | 6624..6956 |
|  |  |  |  |  |
|  | [QIA28738.1](https://www.ncbi.nlm.nih.gov/protein/1808644093) | 6953..7255 | Hypothetical protein | 6953..7255 |
|  | [QIA28739.1](https://www.ncbi.nlm.nih.gov/protein/1808644094) | 7255..7602 | phi 11 orf37 homolog [SA bacteriophages 11, Mu50B] | 7255..7602 |
|  |  |  |  |  |
|  | [QIA28740.1](https://www.ncbi.nlm.nih.gov/protein/1808644095) | 7614..7997 | phi 11 orf38 homolog [SA bacteriophages 11, Mu50B] | 7614..7997 |
|  |  |  |  |  |
|  | [QIA28741.1](https://www.ncbi.nlm.nih.gov/protein/1808644096) | 8016..8597 | Phage tail tube protein | 8016..8597 |
|  | [QIA28742.1](https://www.ncbi.nlm.nih.gov/protein/1808644097) | 8659..9024 | Phi 11 orf40 homolog [SA bacteriophages 11, Mu50B]" | 8659..9024 |
|  |  |  |  |  |
|  | [QIA28743.1](https://www.ncbi.nlm.nih.gov/protein/1808644098) | 9054..9398 | Phi 11 orf41 homolog [SA bacteriophages 11, Mu50B]" | 9054..9398 |
|  |  |  |  |  |
| *Staphylococcus* phage JD419  (MT899504.1) | [QOI66719.1](https://www.ncbi.nlm.nih.gov/protein/1914791446) | Complement  (33156..33455) | Phage protein | Complement (33156..33455) |
| (Uroviricota; *Caudoviricetes; Triavirus*) | [QOI66720.1](https://www.ncbi.nlm.nih.gov/protein/1914791447) | Complement (33501..33665) | Hypothetical protein, phi-ETA orf58 homolog [SA bacteriophages 11, Mu50B] | Complement (33501..33665) |
|  |  |  |  |  |
|  | [QOI66721.1](https://www.ncbi.nlm.nih.gov/protein/1914791448) | Complement (33658..34047) | Phage protein | Complement (33658..34047) |
|  | [QOI66722.1](https://www.ncbi.nlm.nih.gov/protein/1914791449) | Complement (34047..35513) | Phage protein |  |
|  | [QOI66723.1](https://www.ncbi.nlm.nih.gov/protein/1914791450) | Complement (37439..37729) | Hypothetical protein within a prophage | Complement (37439..37729) |

CDS – Coding Sequence

Phages names appear according to their designations presented in the NCBI GenBank database.

**Table S3.** The strain designation, the GenBank accession number, the genome size (bps), the isolation source and country, the collection date, and the PhageAI-determined life cycle, for the phages involved in LGT events

| **Phage designation and taxonomic status** | **Phage GenBank accession #** | **Genome size**  **(bps)** | **Isolation source/actual host species** | **Reference** | **Phage life cycle determined by PhageAI**  **(Prediction accuracy %)** |
| --- | --- | --- | --- | --- | --- |
| *Escherichia* phage vB_EcoM_DE7  (*Caudoviricetes*; *Ounavirinae*; *Felixounavirus*) | OL825705.1 | 86130 | *E. coli* | Cui et al, 2022 | Virulent  (99.17) |
| *Listeria* phage LMSP-25  (*Caudoviricetes; Herelleviridae Jasinkavirinae*; *Pecentumvirus*) | NC_024360.1 | 138036 | *L. monocytogenes* | Unpublished | Virulent  (93.69) |
| *Listeria* phage LMTA-34  (*Caudoviricetes; Herelleviridae Jasinkavirinae; Pecentumvirus*) | NC_042048.1 | 138036 | *L. monocytogenes* | Unpublished | Virulent  (93.69) |
| *Listeria* phage LMTA-57  (*Caudoviricetes; Herelleviridae Jasinkavirinae; Pecentumvirus*) | NC_047871.1 | 136589 | *L. monocytogenes* | Unpublished | Virulent  (93.81) |
| *Salmonella* phage VSe11  (*Caudoviricetes; Ounavirinae*; *Felixounavirus*) | MG251391.1 | 86360 | Sewage/*S. enterica* | Volozhantsev et al, 2018 | Virulent  (98.98) |
| *Salmonella* phage vB_SPuM_SP116  (*Caudoviricetes;Ounavirinae; Felixounavirus*; *Felixounavirus*) | NC_027329.1 | 87510 | Sewage/*S. enterica* | Bao et al. 2019 | Virulent  (99.26) |
| *Campylobacter* phage vB_CjeM_Los1  (*Caudoviricetes;Eucampyvirinae*; *Fletchervirus*) | NC_041896.1 | 134073 | Poultry fecal matter/*C. jejuni* | O'Sullivan et al, 2018 | Temperate  (88.82) |
| *Campylobacter* phage CPt10  (*Caudoviricetes*; *Eucampyvirinae*; *Firehammervirus*) | NC_027996.1 | 175720 | *C. jejuni* | Timms et al. 2010 | Temperate  (85.7) |
| *Staphylococcus* phage SA75  (*Caudoviricetes; Azeredovirinae*; *Dubowvirus*) | MT013111.1 | 43134 | Goat feces/*S. aureus* | D'Souza et al. 2020 | Temperate  (99.25) |
| *Staphylococcus* phage JD419  (Uroviricota; *Caudoviricetes; Triavirus*) | MT899504.1 | 45509 | sputum/*S. aureus* | Feng et al. 2020 | Temperate  (98.96) |
| *Staphylococcus* phage ECel-2020f  (*Uroviricota; Caudoviricetes; Triavirus*) | CP062442.1 | 45361 | N/A | N/A | Temperate  (99.7) |
| *Staphylococcus* phage SA97  (*Caudoviricetes; Azeredovirinae; Dubowvirus*) | NC_029010.1 | 40592 | *S. aureus* | Chang et al. 2015 | Temperate  (99.15) |

Phages names appear according to their designations presented in the NCBI GenBank database.

**Table S4.** The prophage DNA sequences determined by PHASTER across the targeted loci of the bacterial strains involved in LGT

|  |  |  | **PHASTER-generated results** | | | | |
| --- | --- | --- | --- | --- | --- | --- | --- |
| **Organism**  **(GenBank acc #)** | **Genome coordinates for a genetic locus analyzed in recombination analyses (bp)** | **Chromosomal region length**  **(kb)** | **Score** | **Region position in a chromosome** | **Completeness** | **Region GC%** | **Most common phage** |
| *L. monocytogenes*  strain NH1  (CP021325.1) | 675944..676551  (608) | 36.3 | 110 | 669954..706293 | Intact | 36.36 | *Listeria* phage A006  (NC_009815.1) |
|  |  |  |  |  |  |  |  |
| *L. monocytogenes*  strain L2074  (CP007689.1) | 2614217..2614900  (684) | 48.6 | 84 | 2579175-2627817 | Questionable | 36.82 | *Listeria* phage A006  (NC_009815.1) |
|  |  |  |  |  |  |  |  |
| *L. monocytogenes*  strain NCTC7974  (LR134403.1) | 197989..198400  (412) * | 43.5 | 140 | 189306…232883 | Intact | 35.02 | *Listeria* phage LP-101  (NC_024387.1) |
|  |  |  |  |  |  |  |  |
|  |  |  |  |  |  |  |  |
| *C. jejuni*  strain CJ515CC45  (CP012210.1) | 635716..636176  (461) | 13.6 | 20 | 633384..647022 | Incomplete | 25.15 | *Salmonella* phage vB_SosS_Oslo  (NC_018279.1) |
|  |  |  |  |  |  |  |  |
|  |  |  |  |  |  |  |  |
| *S. aureus*  Strain NCCP14558  (CP013953.1) | 1165446..1192496  (27051) | 64.7 | 140 | 1141174..1205962 | Intact | 33.67 | *Staphylococcus* phage SA12  (NC_021801.1) |
|  |  |  |  |  |  |  |  |
| *S. aureus*  strain BPH2869  (LR027869.1) | 1999503..2025595  (26093) | 62.4 | 120 | 1990467..2052921 | Intact | 33.89 | *Staphylococcus* phage 53  (NC_007049.1) |
|  |  |  |  |  |  |  |  |
| *S. aureus*  strain ER01116.3  (CP030516.1) | 1975903..2001993  (26091) | 59.1 | 130 | 1974750..2033893 | Intact | 33.73 | *Staphylococcus* phage 53  (NC_007049.1) |
|  |  |  |  |  |  |  |  |
| *S. aureus*  strain NCTC13140  (LS483319.1) | 2028541..2054630  (26090) | 45.2 | 60 | 2027356..2072582 | Incomplete | 34.44 | *Staphylococcus* phage 53  (NC_007049.1) |
|  |  |  |  |  |  |  |  |
| *S. aureus*  strain ER02836.3  (CP030432.1) | 1228856..1254950  (26095) | 43.5 | 60 | 1213284..1256834 | Incomplete | 34.08 | *Staphylococcus* phage 53  (NC_007049.1) |
|  |  |  |  |  |  |  |  |
| *S. aureus*  strain MRSA107  (CP018629.1) | 910419..920451  (10033) | 71.9 | 130 | 859860..931799 | Intact | 32.85 | *Staphylococcus* phage YMC/09/04/R1988  (NC_022758.1) |
| *S. aureus*  Strain N17CSA11  (CP107527.1) | 425766..435798  (10033) | 62.6 | 150 | 418411..481069 | Intact | 32.89 | *Staphylococcus* phage YMC/09/04/R1988  (NC_022758.1) |
|  |  |  |  |  |  |  |  |
| *S. aureus*  strain FDAARGOS_1  (CP026968.1) | 213079..223050  (9972) | 49.4 | 110 | 174025..223478 | Intact | 33.83 | *Staphylococcus* phage YMC/09/04/R1988  (NC_022758.1) |
|  |  |  |  |  |  |  |  |
| *S. aureus*  strain 14640  (CP053636.1) | 1238916..1248887  (9972) | 57.3 | 120 | 1231755..1289105 | Intact | 33.11 | *Staphylococcus* phage YMC/09/04/R1988  (NC_022758.1) |
| *S. aureus* strain ER01174.3  (CP030712.1) | 1523927..1533958  (10032) | 61.6 | 150 | 1516571..1578184 | Intact | 32.89 | *Staphylococcus* phage phi2958PVL  (NC_011344.1) |

197989..198400 (412) * - *L. monocytogenes* Plasmid 6 region

Phages names appear according to their designations presented in the NCBI GenBank database.

**Table S5.** The results of the RDP4 analyses exhibiting the recombination beginning and end breakpoints across the LGT-affected genetic loci, and the trajectories of the LGT events of these loci, involving *Staphylococcus* phages SA75 and JD419, and the *S. aureus* strains

| **Recombinant strain**  **(GenBank acc. #)** | | **Major donor**  **(GenBank acc. #)** | | **Minor donor**  **(GenBank acc. #)** | **CDS, for a protein, within a phage genomic region examined**  **(Coordinates in a phage genome [size in bps])** | **Recombination beginning and end breakpoints**  **(99% Cl)** | | ***P*-value generated by**  **the RDP4 algorithm** | |
| --- | --- | --- | --- | --- | --- | --- | --- | --- | --- |
| *Staphylococcus* phage SA75 (MT013111.1) | | *S. aureus* strain NCCP14558  (CP013953.1) | *S. aureus* strain BPH2869  (LR027869.1) | Putative major teichoic acid biosynthesis protein C (ID: [QIA28747.1](https://www.ncbi.nlm.nih.gov/protein/1808644102)) CDS: 15764..17674.  (1..27050) | 16406 (16289-16506) – 17310 (17277-17328) | RDP:  GENECONV:  BootScan:  MaxChi:  Chimaera:  SiScan:  3Seq: | | 2.711 x 10^-81^  8.243 x 10^-77^  3.583 x 10^-80^  9.110 x 10^-23^  1.819 x 10^-22^  1.375 x 10^-24^  1.243 x 10^-14^ |  |
|  | |  |  |  |  |  | |  |  |
| *Staphylococcus* phage SA75 (MT013111.1) | | *S. aureus* strain NCCP14558  (CP013953.1) | Unknown | CDS of hypothetical proteins (IDs: [QIA28737.1](https://www.ncbi.nlm.nih.gov/protein/1808644092); [QIA28738.1](https://www.ncbi.nlm.nih.gov/protein/1808644093); [QIA28739.1](https://www.ncbi.nlm.nih.gov/protein/1808644094); [QIA28740.1](https://www.ncbi.nlm.nih.gov/protein/1808644095); [QIA28741.1](https://www.ncbi.nlm.nih.gov/protein/1808644096);  [QIA28742.1](https://www.ncbi.nlm.nih.gov/protein/1808644097); [QIA28743.1](https://www.ncbi.nlm.nih.gov/protein/1808644098)) respectively: 6624..6956; 6953..7255; 7255..7602; 7614..7997; 8016..8597; 8659..9024; 9054..9398. Tape measure protein (ID: [QIA28744.1](https://www.ncbi.nlm.nih.gov/protein/1808644099)) CDS: 9415..12879.  Putative distal tail protein (ID: [QIA28745.1](https://www.ncbi.nlm.nih.gov/protein/1808644100)) CDS: 12892..13839. Putative tail associated lysin (ID: [QIA28746.1](https://www.ncbi.nlm.nih.gov/protein/1808644101)) CDS: 13848..15749.  (1..27050) | 7166 (6662-7184) – Undetermined (10312-14443) | RDP:  GENECONV:  BootScan:  MaxChi:  Chimaera:  SiScan:  3Seq: | | 7.617 x 10^-48^  1.114 x 10^-42^  8.180 x 10^-43^  6946 x 10^-22^  3.726 x 10^-25^  5.539 x 10^-21^  6.217 x 10^-15^ |  |
|  | |  |  |  |  |  | |  |  |
| *Staphylococcus* phage SA75 (MT013111.1) | | *S. aureus* strain NCCP14558  (CP013953.1) | Unknown | Lysin (ID: [QIA28752.1](https://www.ncbi.nlm.nih.gov/protein/1808644107)) CDS: 20528..22426.  (1..27050) | 21478 (21395-21552) – 21940 (21893-21993) | RDP:  GENECONV:  BootScan:  MaxChi:  Chimaera:  SiScan:  3Seq: | | 1.453 x 10^-20^  2.115 x 10^-19^  1.473 x 10^-20^  6.104 x 10^-07^  5.606 x 10^-07^  8.351 x 10^-09^  8.393 x 10^-13^ |  |
|  | |  |  |  |  |  | |  |  |
| *S. aureus* strain BPH2869  (LR027869.1) | | *S. aureus* strain ER01116.3  (CP030516.1) | *Staphylococcus* phage SA75 (MT013111.1) | Lysin (ID: [QIA28756.1](https://www.ncbi.nlm.nih.gov/protein/1808644111)) CDS: 24487..25932.  (1..27050) | Undetermined (25461-25528) – Undetermined (25578-25741) | RDP:  GENECONV:  BootScan:  MaxChi:  Chimaera:  SiScan:  3Seq: | | 8.090 x 10^-12^  6.256 x 10^-11^  6.941 x 10^-12^  3.389 x 10^-03^  4.002 x 10^-02^  -  1.653 x 10^-07^ |  |
|  | |  |  |  |  |  | |  |  |
| *S. aureus* strain ER01116.3  (CP030516.1) | | *S. aureus* strain NCTC13140  (LS483319.1) | *Staphylococcus* phage SA75 (MT013111.1) | Hypothetical protein (ID: [QIA28751.1](https://www.ncbi.nlm.nih.gov/protein/1808644106)) CDS: 20092..20391. Lysin (ID: [QIA28752.1](https://www.ncbi.nlm.nih.gov/protein/1808644107)) CDS: 20528..22426.  (1..27050) | 20316 (20195-20349) – 20424 (20350-21230) | RDP:  GENECONV:  BootScan:  MaxChi:  Chimaera:  SiScan:  3Seq: | | 3.017 x 10^-07^  1.967 x 10^-05^  -  6.603 x 10^-04^  3.272 x 10^-04^  -  7.630 x 10^-04^ |  |
|  | |  |  |  |  |  | |  |  |
| *S. aureus* strain ER01116.3  (CP030516.1) | | *S. aureus* strain NCTC13140  (LS483319.1) | *Staphylococcus* phage SA75 (MT013111.1) | Putative tail associated lysin (ID: [QIA28746.1](https://www.ncbi.nlm.nih.gov/protein/1808644101))CDS: 13848..15749. Putative major teichoic acid biosynthesis protein C (ID: [QIA28747.1](https://www.ncbi.nlm.nih.gov/protein/1808644102)) CDS: 15764..17674.  (1..27050) | 14914 (14790-15247) – 15100 (14790-15247) | RDP:  GENECONV:  BootScan:  MaxChi:  Chimaera:  SiScan:  3Seq: | | -  -  5.638 x 10^-03^  2.599 x 10^-04^  -  -  1945 x 10^-02^ |  |
|  | |  |  |  |  |  | |  |  |
| *S. aureus* strain ER01116.3  (CP030516.1) | | *S. aureus* strain NCTC13140  (LS483319.1) | *Staphylococcus* phage SA75 (MT013111.1) | Hypothetical protein (ID: [QIA28749.1](https://www.ncbi.nlm.nih.gov/protein/1808644104))  CDS: 19497..19874; CDS of hypothetical proteins (IDs: [QIA28750.1](https://www.ncbi.nlm.nih.gov/protein/1808644105); [QIA28751.1](https://www.ncbi.nlm.nih.gov/protein/1808644106)) respectively: 19875..20051; 20092..20391.  (1..27050) | Undetermined (19704-20005) – Undetermined (20016-20136) | RDP:  GENECONV:  BootScan:  MaxChi:  Chimaera:  SiScan:  3Seq: | | -  1.954 x 10^-05^  -  -  -  -  1.283 x 10^-02^ |  |
|  | |  |  |  |  |  | |  |  |
| *Staphylococcus* phage JD419  (MT899504.1) | | *S. aureus* strain MRSA107  (CP018629_1) | *S. aureus* strain N17CSA11  (CP107527.1) | Lysin (ID: [QOI66741.1](https://www.ncbi.nlm.nih.gov/protein/1914791468)) CDS: 759..2213. Holin (ID: [QOI66742.1](https://www.ncbi.nlm.nih.gov/protein/1914791469)) CDS: 2225..2527.  Hypothetical protein (ID: [QOI66719.1](https://www.ncbi.nlm.nih.gov/protein/1914791446)) CDS: 2663..2962. Hypothetical protein (ID: [QOI66720.1](https://www.ncbi.nlm.nih.gov/protein/1914791447)) CDS: 3008..3172. Hypothetical protein (ID: [QOI66721.1](https://www.ncbi.nlm.nih.gov/protein/1914791448)) CDS: 3165..3554. Hypothetical protein (ID: [QOI66722.1](https://www.ncbi.nlm.nih.gov/protein/1914791449) ) CDS: 3554..5020. Putative major teichoic acid biosynthesis protein C (ID: [QOI66743.1](https://www.ncbi.nlm.nih.gov/protein/1914791470)) CDS: 5020..6930. Hypothetical protein (ID: [QOI66723.1](https://www.ncbi.nlm.nih.gov/protein/1914791450))CDS: 6946..7236. Tail fiber (ID: [QOI66744.1](https://www.ncbi.nlm.nih.gov/protein/1914791471)) CDS: 7236..8819. Tail fiber (ID: [QOI66745.1](https://www.ncbi.nlm.nih.gov/protein/1914791472))  CDS: 8828..9652. Tail length tape-measure protein (ID: [QOI66746.1](https://www.ncbi.nlm.nih.gov/protein/1914791473)) CDS: 9652..>10032.  (30494..40525) | 3364 (2808-3620) – 10025 (10013-32) | RDP:  GENECONV:  BootScan:  MaxChi:  Chimaera:  SiScan:  3Seq: | | -  2.610 x 10^-35^  2.796 x 10^-12^  1.382 x 10^-26^  1.043 x 10^-26^  1.026 x 10^-22^  3.667 x 10^-74^ |  |

CDS - Coding Sequence

Phages names appear according to their designations presented in the NCBI GenBank database.

Recombination beginning and end breakpoints (99% Cl) * - The recombination beginning and end breakpoints in the DNA alignment
